# Electron Microscopy Transfer System to Protect Atmosphere‐Sensitive Materials for Scanning Electron Microscopy Characterization

**DOI:** 10.1002/jemt.70107

**Published:** 2025-12-31

**Authors:** Louis G. Corcoran, Ellen M. Monzo, Chinomso E. Onuoha, Shivasheesh Varshney, Han Seung Lee, Chris Frethem, Bharat Jalan, Alon V. McCormick, R. Lee Penn

**Affiliations:** ^1^ Department of Chemistry University of Minnesota Minneapolis Minnesota USA; ^2^ Department of Chemical Engineering and Materials Science University of Minnesota Minneapolis Minnesota USA; ^3^ Characterization Facility University of Minnesota Minneapolis Minnesota USA

**Keywords:** atmosphere‐sensitive materials, hygroscopic materials, sample transfer system, scanning electron microscopy

## Abstract

Atmosphere‐ and/or moisture‐sensitive materials can be challenging to characterize using electron microscopy techniques due to sample preparation workflows that generally require exposure to ambient conditions. Here, we describe a novel preparation method that uses aluminum foil in combination with a commercial cryo‐EM transfer system to circumvent undesired exposure to the atmosphere. First, hygroscopic MgCl_2_ was used as a model material, and prepared samples (both protected and unprotected) were placed in a controlled‐humidity environment (> 80% relative humidity) for various exposure lengths (circa seconds to hours). Following this, the effectiveness of the sample preparation method was determined by comparing qualitative photos and quantitative X‐ray diffraction patterns between the two sample subsets. The combined results of these experiments suggest that the outlined preparation method effectively protects MgCl_2_ from atmospheric contamination compared to MgCl_2_ samples that had no protective measures taken. Finally, the preparation method was utilized to protect a highly hygroscopic crystalline BaO thin film for characterization via scanning electron microscopy, thereby demonstrating a functional application of the outlined preparation technique and an additional use for the commercial cryo‐EM transfer system beyond its intended application.

## Highlights

- SEM–EDX and XRD characterization confirm that pouches made from readily available aluminum foil effectively protect highly atmosphere‐sensitive materials from contamination during transfer from inert environments to the SEM.
- Cryo‐EM transfer system features an integrated workflow and toolset that enables the successful deprotection of atmosphere‐sensitive materials under inert conditions, followed by their transfer into the high‐vacuum SEM environment.

## Graphical abstract

SEM–EDX and XRD characterization confirm that pouches made from readily available aluminum foil, used in combination with a commercial cryo‐EM transfer system, effectively protect highly atmosphere‐sensitive and/or hygroscopic materials from contamination during transfer from inert environments to the SEM vacuum chamber.

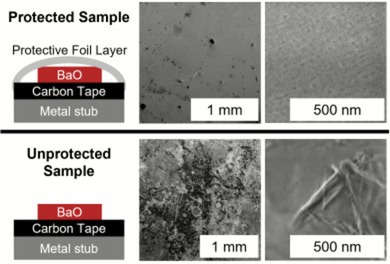

## 1 Introduction

Scanning electron microscopy (SEM) has notable limitations that can impact its effectiveness for the characterization of microstructure, chemical composition, and nanoscale features of samples. For instance, samples may degrade rapidly under the electron beam, or samples may be sensitive to temperature, vacuum conditions, or moisture. To address these challenges, specialized instrument capabilities, such as variable‐pressure and environmental SEM, and advanced preparation techniques have been developed to enable the characterization of otherwise problematic materials (Gaume and Joubert 2011; Redzic et al. 2016; Baudry et al. 1988; Hovington et al. 2014; Simon et al. 2017; Orsini et al. 1998).

Characterizing air‐sensitive materials (e.g., hygroscopic, deliquescent, and/or pyrophoric materials) can be challenging because these materials are typically highly reactive and can easily be contaminated or damaged when exposed to air. Additionally, the workflow from lab preparation to the instrument includes difficult‐to‐avoid exposure to the ambient atmosphere. Some examples of relevant materials are ceramics including SrI_2_ (used in or being developed for a variety of applications including scintillators for atmospheric/environmental radiation detection (Boatner et al. 2013; Czyz et al. 2021; Kowatari et al. 2019) and medical imaging (Matei et al. 2020)), perovskites for photovoltaic applications (Yuce et al. 2022; Zhang et al. 2018, 2020; Leguy et al. 2015; Christians et al. 2015), metallic Li deposition/dissolution onto electrolytes for Li‐ion batteries (Chen et al. 2011; Langenhuizen 1998), MgCl_2_ as a support for Ziegler–Natta catalysts (Redzic et al. 2016; Andoni et al. 2008; Yu et al. 2019), and alkaline earth metal‐based oxides which are used as sacrificial layers to exfoliate functional thin films, allowing their heterogeneous integration with incompatible material systems (Varshney et al. 2024). In the case of alkaline earth metals (*X* = Mg, Ca, Sr, and Ba), they readily form corresponding hydroxides (*X*(OH)_2_) upon contact with moisture in air (Varshney et al. 2024). To illustrate this further, ceramics such as SrI_2_, BaO, and Ba_3_Al_2_O_6_ have been shown to develop detrimental defects with less than 10 s of air exposure (Gaume and Joubert 2011; Varshney et al. 2024; Takahashi and Lippmaa 2020; Singh et al. 2019). Bulk anhydrous MgCl_2_ can transform into a hydrated form in as little as 3 min in a humid environment (Figure S1). Similarly, Li‐containing materials undergo reactions with molecules such as nitrogen, oxygen, and water, which means there exists a significant risk of contamination and damage after even brief exposure to air (Simon et al. 2017; Chen et al. 2011; Langenhuizen 1998; Phillips and Tanski 2005). The perovskite CH_3_NH_3_PbI_3_ holds promise for next‐generation solar cell applications but humidity exposure during processing may catalyze material decomposition (Frost et al. 2014) or result in a morphological change in the material (Leguy et al. 2015; Christians et al. 2015; Eperon et al. 2014).

Thus, the paucity of good sample preparation methods has been recognized, and previous work has made progress on this front. Gaume and Joubert (2011) developed an air‐tight apparatus that was sealed with an elastic membrane and could be transported from a protected sample preparation environment (viz. glovebox) to the instrument; however, this system requires the use of a variable pressure SEM and the custom fabrication of the device. Other methods include transporting samples (from glovebox to instrument) in sealed aluminum‐thermoplastic laminate bags (Baudry et al. 1988; Hovington et al. 2014) or Mylar films (Redzic et al. 2016), the design of a self‐sealing transfer box (Simon et al. 2017), and elaborate sample transfer attachment systems (Orsini et al. 1998). Some of these approaches, while simple, still result in the exposure of the sample to the ambient environment for short periods of time (Redzic et al. 2016; Baudry et al. 1988) and therefore may not provide adequate protection for a given material. On the other hand, options like the self‐sealing transfer box and sample transfer attachment system require the machining of complex ancillary tools (Simon et al. 2017) or specially designed systems to successfully protect the sample (Orsini et al. 1998). Further, most of these approaches do not appear to be conducive to use with materials that might require additional preparation steps (e.g., sputter coating), either limiting sample applicability/instrumental capabilities or increasing sample exposure time to ambient conditions (i.e., transfer into/out of sputter coater instrument and subsequent transfer into SEM, all with exposure to ambient conditions).

In this study, we present a sample preparation method that prevents air and moisture from coming into contact with the sample material. Our method features commercially available sample preparation instrumentation along with common and inexpensive laboratory supplies, and we use hygroscopic alkaline earth metal‐based ceramics, namely MgCl_2_ and BaO, to demonstrate technique efficacy. MgCl_2_ was an excellent proof‐of‐concept candidate for this study because the effectiveness of sample protection could be readily verified with powder x‐ray diffraction (XRD) diffractograms with bulk samples. To probe the efficacy of our method more sensitively, we synthesized a single‐crystalline BaO thin film sample using molecular beam epitaxy (MBE). BaO is often used as a sacrificial layer for membrane fabrication processes where nanometer‐scale layers have been shown to completely dissolve in water in less than 10 s (Takahashi and Lippmaa 2020)—we utilize it here for SEM characterization due to its high sensitivity to moisture, since even minor exposure to atmospheric water should cause significant and observable surface degradation of the material. Finally, the method presented herein can be readily modified to accommodate the properties of any given material of interest.

## 2 Materials and Methods

### 2.1 MgCl_2_ Proof‐of‐Concept Experiments

Detailed schematics of all procedures outlined within this section can be found in Schemes S1–S4.

#### 2.1.1 MgCl_2_ Preparation

Roughly 5 g of MgCl_2_ (Sigma Aldrich, anhydrous ≥ 98% purity) was placed in a 20 mL borosilicate scintillation vial (Duran Wheaton Kimble), capped with aluminum foil, and purified in a vacuum oven (Yamato Scientific Co). The sample was placed in the oven at room temperature and heated to 235°C at a pressure of ~1.3 kPa for at least 15 h before turning the oven off. While under vacuum, the oven was allowed to cool to 50°C or below before releasing the vacuum and extracting the scintillation vial. Upon extraction, the vial was immediately capped (Supelco, PTFE‐lined), wrapped in parafilm, and moved into a vacuum desiccator for later use. A small mass of this sample was reserved as a standard (“MgCl_2_ Standard”; Figures 4 and 5) and was not manipulated further.

#### 2.1.2 Sample Preparation for Characterization

**FIGURE 1:**
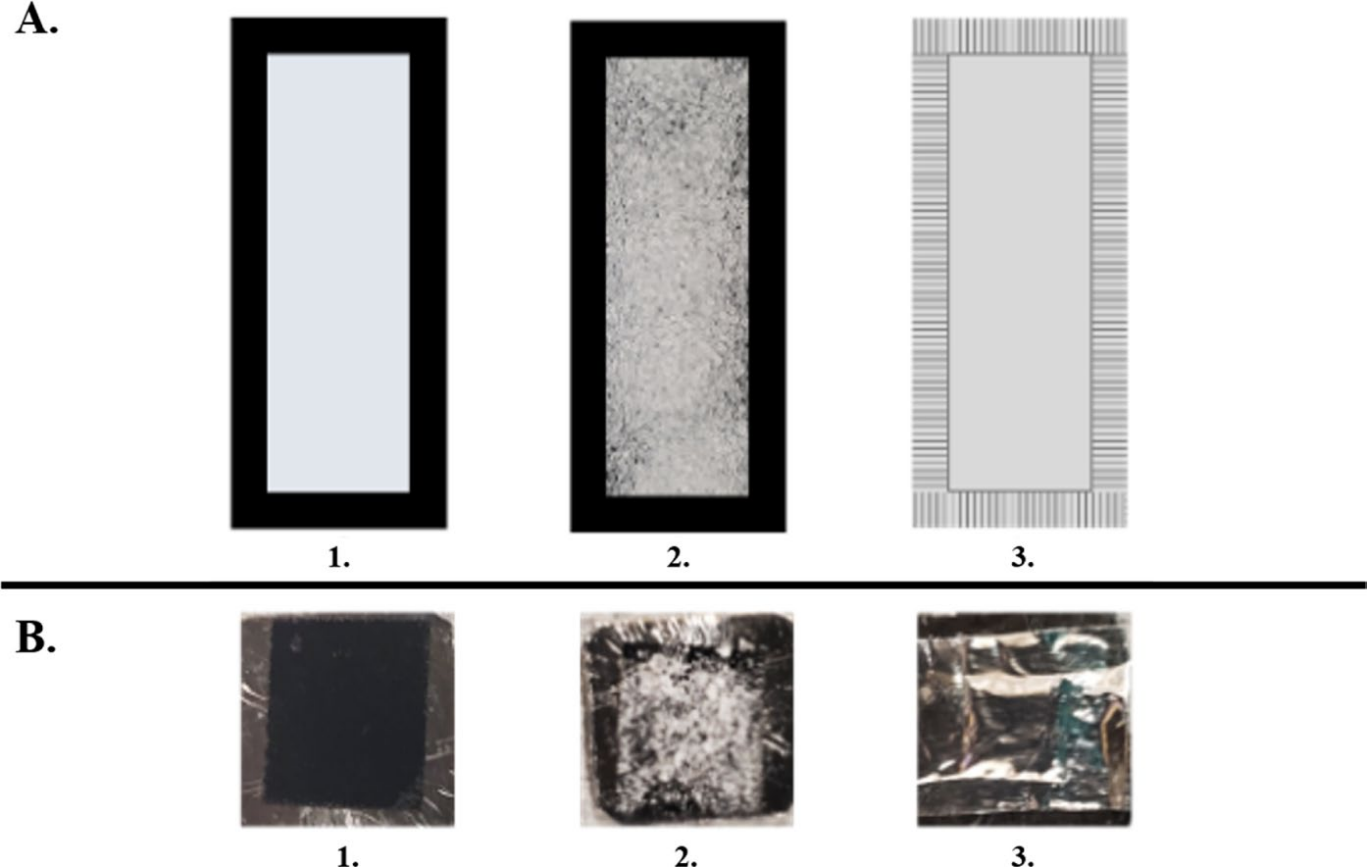
(A) Sample preparation schematic for the protected MgCl_2_ on glass slides. 1. Glass slide with carbon tape adhered to the outside edges of the slide. 2. MgCl_2_ spread evenly across the glass slide. 3. Aluminum foil pressed firmly to the carbon tape over the MgCl_2_ sample. (B) Sample preparation for the protected MgCl_2_ on metal stubs. 1. Example metal stub with carbon tape affixed to the stub. 2. MgCl_2_ salt spread evenly in the center of the carbon tape. 3. Aluminum foil pressed firmly to the carbon tape over the MgCl_2_ sample.

MgCl_2_ was removed from the vacuum desiccator, and the cap was removed. Immediately after removing the cap, the vial and cap were placed in the antechamber of a dry glovebox (Labconco), and the vacuum was pulled for 4 min before refilling the antechamber with N_2_ gas (ultrahigh purity, Grade 5); once the pressure in the antechamber reached 0 in Hg, the vacuum was again pulled for 4 min. This process was repeated for four cycles before the sample was moved into the glovebox. The humidity on the day of sample transfer into the box was < 30% relative humidity (RH; Fisherbrand Traceable). The following materials were also transferred to the glovebox: 10 plastic weigh boats, 5 glass microscope slides (Fisher Scientific; cleaned with methanol and a kimwipe and dried in a glassware oven at 50°C for 5–10 min) with carbon tape affixed to all four edges (Figure 1A1; Note that the carbon tape overlapped in the corners and the tape backing was kept on the tape, except in the corners, to prevent debris from adsorbing to the tape), loosely folded aluminum foil (~900 cm^2^; Clark FoodService Inc. Stock #CF 1851), a small pair of scissors, and six metal rectangle stubs with dimensions ~1 cm^2^ × 1 mm thick (Figure 1B1); one side of each metal stub was covered with carbon tape with the backing kept on.

#### 2.1.3 Glass Slide and Weigh Boat Preparation

In the glovebox, 0.15 g of MgCl_2_ was massed on a tared plastic weigh boat for sample preparation (Mettler Toledo AG104); 10 such samples were prepared in succession. For the first five, the MgCl_2_ was transferred to one of the glass slides (the tape backing was removed prior to salt transfer) and spread evenly across the slide in a thin layer with a cleaned metal spatula (Figure 1A2). A rectangular strip of aluminum foil, slightly larger than the dimensions of the whole glass slide, was then cut with the scissors and applied to the tape over the MgCl_2_ sample with a clean foil surface covering the sample (taking care to press the foil firmly and evenly across the tape edges surrounding the sample using metal tweezers and a flexible, rounded metal spatula; Figure 1A3).

#### 2.1.4 Metal Stub Preparation—MgCl_2_

After removing the carbon tape backing from the stub, enough MgCl_2_ to lightly cover ~75% of the carbon tape when pressed/spread into the tape was transferred to the center of the carbon tape with a metal spatula and spread thinly (Figure 1B2). A strip of foil slightly larger than the metal surface was then cut and applied over the sample. The edges of the foil were then crimped on the tape edges surrounding the sample using metal tweezers and a flexible, rounded metal spatula (taking care not to rip the foil protecting the sample; Figure 1B3).

#### 2.1.5 Controlled Humidity Experiments

A 1 L beaker with DI water was placed in a sealed plexiglass box (100 × 70 × 55 cm^3^ *L* × *W* × *H*) with a small gasketed door. A glass gas dispersion tube (ASTM 10–20 μL) with air flowing through it was placed inside the beaker; after several hours, the internal RH was measured at 90%+ with a Fisherbrand Traceable digital thermometer (hereafter, this will be referred to as the humidity box [HB]). Following this, the samples prepared in the glovebox were taken out and placed in the HB. The door was closed; the process for the samples prepared on metal stubs will be discussed first.

The samples prepared on the small metal stubs were used to collect qualitative proof‐of‐concept data. The salt used to prepare these samples could not be characterized via XRD due to the small volume of MgCl_2_ used and its subsequent adhesion to carbon tape. Instead, the experiments detailed herein were conducted to visualize the effectiveness of sample protection and/or the extent of hydration for MgCl_2_ loadings that would be consistent with SEM characterization. Before placing the stubs in the HB, the foil pouch on one stub was opened with a razor blade to expose the salt to air; this stub was used as the control stub (CS). Following this, all six stubs were then transferred into the HB, and stubs were taken out in succession at 5‐, 15‐, 30‐, 60‐, and 180‐min time points, with the CS taken out at each time point for comparison. Note, during the subsequent sample extraction from the HB, the humidity did fluctuate but never fell below 84% RH. Following extraction, the foil pouch was cut open, and an image was taken of both the protected salt sample and that of the CS with a cell phone camera. The CS was then placed back in the HB and extracted at each subsequent time point to collect a comparison image.

The MgCl_2_ samples prepared on protected glass slides and weigh boats were immediately transferred to the HB upon removal from the glovebox. One of each sample was taken out of the HB at the following time points: 5‐, 15‐, 30‐, 60‐, and 180‐min. At each time point, the two samples were transferred to a labeled 20 mL scintillation vial (dried at 50°C prior) that was then sealed with a cap wrapped in parafilm before storing in a vacuum desiccator. To transfer the sample from the weigh boats, a cleaned metal spatula was used. For samples on the microscope slides, a razor blade was used to cut the foil into a “v” shape and remove the carbon tape from one end of the slide. A clean metal spatula was then used to scrape the salt into the vial. After all samples were extracted from the HB, nine sample vials were transferred to the glovebox (the weigh boat sample exposed to high humidity for 180 min had become a liquid brine, which was unsuitable for the glovebox). To do so, samples were all uncapped and placed in the antechamber of the glove box. Vacuum was immediately pulled for 4 min and cycled with N_2_ gas. Following this, vacuum was then pulled for 2 min and cycled with N_2_ gas; three iterations of this shorter cycle were completed. The samples were then brought into the glovebox and immediately capped and wrapped in parafilm. These samples, along with one control MgCl_2_ standard (stored in the glovebox during HB experiments), were used for the XRD experiments discussed below. Glass‐slide samples prepared in this way were also utilized for mass‐gain experiments to provide further evidence for the effectiveness of the protection protocol; the experimental procedure and the results of these experiments can be found in Supporting Information S1: Section 2.

#### 2.1.6 Powder XRD

To quantify the effectiveness of the sample protection method, direct comparisons were made between the XRD patterns of (1) the control sample “MgCl_2_ standard” and the protected MgCl_2_ samples, (2) the control sample “MgCl_2_ standard” and the exposed MgCl_2_ samples, and (3) the protected and exposed samples across all five time points. To prepare each sample for XRD, a sample holder equipped with a plastic dome (to protect the sample; Anton Paar) was transferred into the glovebox with three well‐fitting, zero background Si (Si [510] zero background insert) sample wafers already placed in the sample stage. These components were cycled into the box using the 4‐, 2‐, 2‐, 2‐min vacuum and nitrogen cycling method described above. Once in the glovebox, enough material from a given sample was transferred with a spatula to the stage to provide a uniform deposit on the Si wafer (enough sample such that the sample height appeared visually level with the well on the sample holder). Following this, the plastic dome was affixed to the sample stage using the tools included in the kit. The entire apparatus was immediately extracted from the box and loaded onto a spinning sample stage apparatus (PANalytical Reflection Transmission Spinner PW3064) in the XRD instrument (PANalytical X'Pert Pro), and the XRD pattern was collected. Diffraction patterns were collected using Co Kα radiation (*λ* = 1.79 Å), using operating conditions of 45 kV and 40 mA. Patterns were collected from 10° to 70° 2*θ* using an effective dwell time of 105 s per step and a step size of 0.0167° 2*θ* for a total scan length of 51.5 min. The scan time was selected to require less than 1 h to ensure that the domed sample holder maintained a sufficient air‐ and moisture‐free environment for the duration of the measurement. A powder XRD pattern of the domed XRD sample holder, along with the three Si background wafers (no sample), was also collected using the same program.

Powder XRD diffraction patterns were simulated from crystallographic information files for MgCl_2_·H_2_O (Sugimoto et al. 2007), MgCl_2_·2H_2_O (Sugimoto et al. 2007), MgCl_2_·4H_2_O (Schmidt et al. 2012), MgCl_2_·6H_2_O (Villars and Cenzual 2016), MgCl_2_·8H_2_O (Hennings et al. 2013), and MgCl_2_·12H_2_O (Hennings et al. 2013) using CrystalDiffract 6 or Mercury 4.1.3 software. These simulated powder patterns were compared against experimental data. Powder XRD file MgCl_2_ 01‐089‐1567 was also used as a reference. Graphs of experimental data plotted against all six reference patterns are included in Figures S2 and S3. Experimental powder XRD patterns displayed a broad amorphous peak centered at ~20.6° 2*θ* due to the plastic dome of the sample holder. To correct for this, the intensity recorded at each 2*θ* value for the plastic dome was subtracted from each corresponding data point in the experimental patterns. The slight downturn in the baseline of experimental patterns at low 2*θ* is an artifact of the point‐by‐point baseline correction.

### 2.2 BaO Thin Film Synthesis

Thin films of BaO grown by MBE were used to further probe the efficacy of the sample preservation method. Epitaxial single‐crystalline 100 nm BaO thin film was grown by MBE by co‐deposition of Barium (Ba) and oxygen plasma on Nb‐doped SrTiO_3_ (001) single crystal substrate (CrysTec GmbH); additional examples of Ba growth via MBE methodology are detailed in the literature (Varshney et al. 2024; Takahashi and Lippmaa 2020; Ohnishi et al. 1997; Takahashi et al. 2002). Two samples were loaded together for the growth of the primary test sample and a reference sample. A 99.99% purity Barium source was supplied using an elemental effusion cell operated at a cell temperature of 563°C, giving the beam equivalent pressure (BEP) of 7.89 × 10^−8^ Torr. Oxygen plasma at 250 W at an oxygen pressure of 5 × 10^−6^ Torr was used to clean the substrate prior to growth for 25 min. The substrate was maintained at a temperature of 600°C for the growth of the BaO film. During growth, reflection high‐energy electron diffraction (RHEED; Staib Instruments) was used to monitor thin film growth. After growth, the sample was transferred into the load lock chamber, which has a base pressure of 5 × 10^−9^ Torr. To take out the sample from the vacuum, an atmosphere‐controlled glovebox filled with N_2_ was connected to the load lock chamber. The load lock was vented with N_2_, and the sample was taken out in an N_2_ glass container attached to the load lock chamber. The samples were then transported in a desiccator to an N_2_ glovebox, where they were prepared for SEM characterization.

### 2.3 BaO Sample Preparation for Characterization via SEM

The BaO‐containing desiccator was first placed in the antechamber of a N_2_ glovebox along with carbon tape, aluminum foil (which had been dried in a vacuum oven at 150°C for 1 h), and two metal stubs (1 cm × 1 cm × 1 mm). Vacuum was then pulled to evacuate ambient air and moisture in the chamber until the vacuum pressure was below −30 in Hg. The antechamber was then filled with N_2_ gas (ultrahigh purity) to a pressure of 0 in Hg vacuum before transferring the desiccator into the glovebox. The moisture level inside the glovebox was < 21 ppm. A double‐sided carbon tape was then fixed onto the metal stub, making sure to cover all four edges. Note that the carbon tape overlapped in the corners, and the tape backing was kept on the tape, except in the corners, to prevent debris from adsorbing to the tape.

After removing the carbon tape backing from the stub, the BaO sample was placed in the center of the tape and pressed lightly. A strip of aluminum foil, slightly larger in area than the metal surface, was then cut and applied to the sample. Using metal tweezers, the edges of the foil surrounding the sample were pressed onto the tape to ensure a tight seal. During sealing, care was taken (1) not to rip the foil and (2) to leave enough room above the sample to prevent it from puncturing the foil. While in the N_2_ glovebox, samples were placed in a sealed plastic container that was transferred back into the desiccator; the entire desiccator assembly was transported to the characterization facility. Two BaO sample sets were prepared in this manner; one sample was not sputter‐coated, while the second was coated with 2 nm platinum to demonstrate the ability to sputter coat samples with this method (Supporting Information S1: Section 3). Both samples were characterized using SEM before and after exposure to controlled humidity. A detailed flowchart outlining the steps in this section, as well as those outlined in Section 2.4, can be found in Scheme S5.

### 2.4 SEM

**FIGURE 2:**
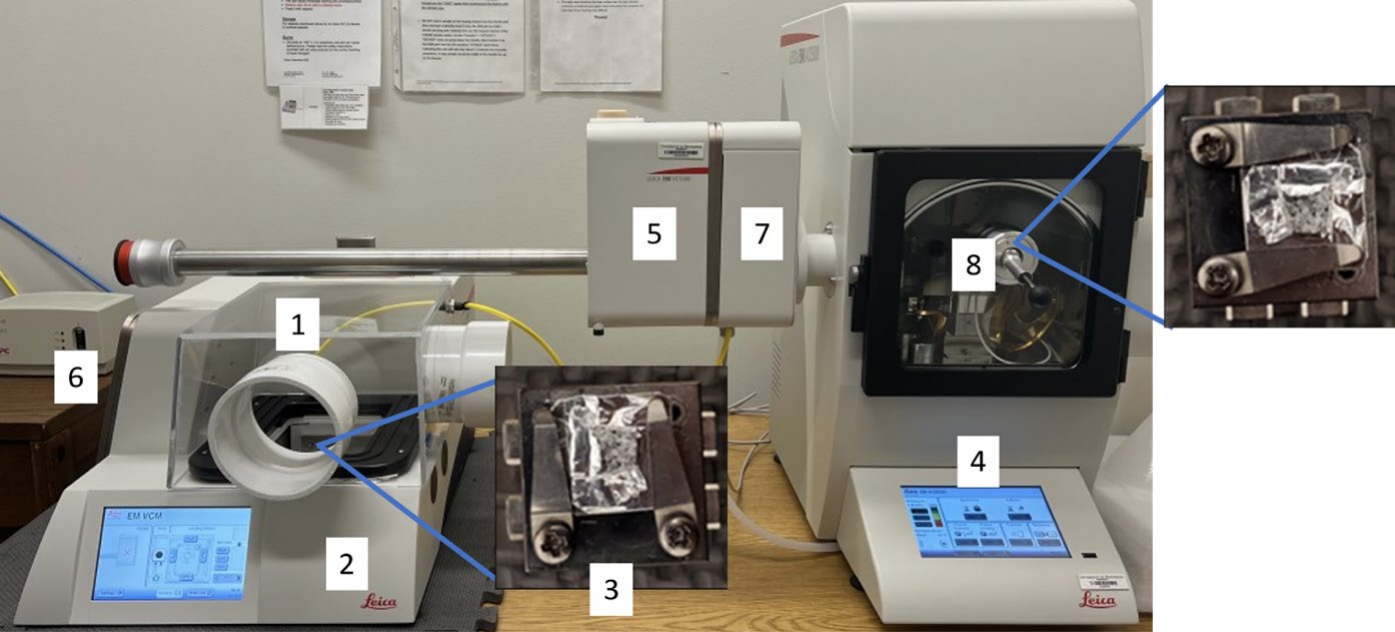
SEM workflow from protected metal stub through sputter coating. Image includes plexiglass box (1), Leica EM VCM (2), metal stub affixed to SEM stub (3), Leica EM ACE600 sputter coater (4), Leica EM VCT500 (5), exchange port on VCM (6), Leica EM ACE600 (7), and knife to open foil pouch (8).

**FIGURE 3:**
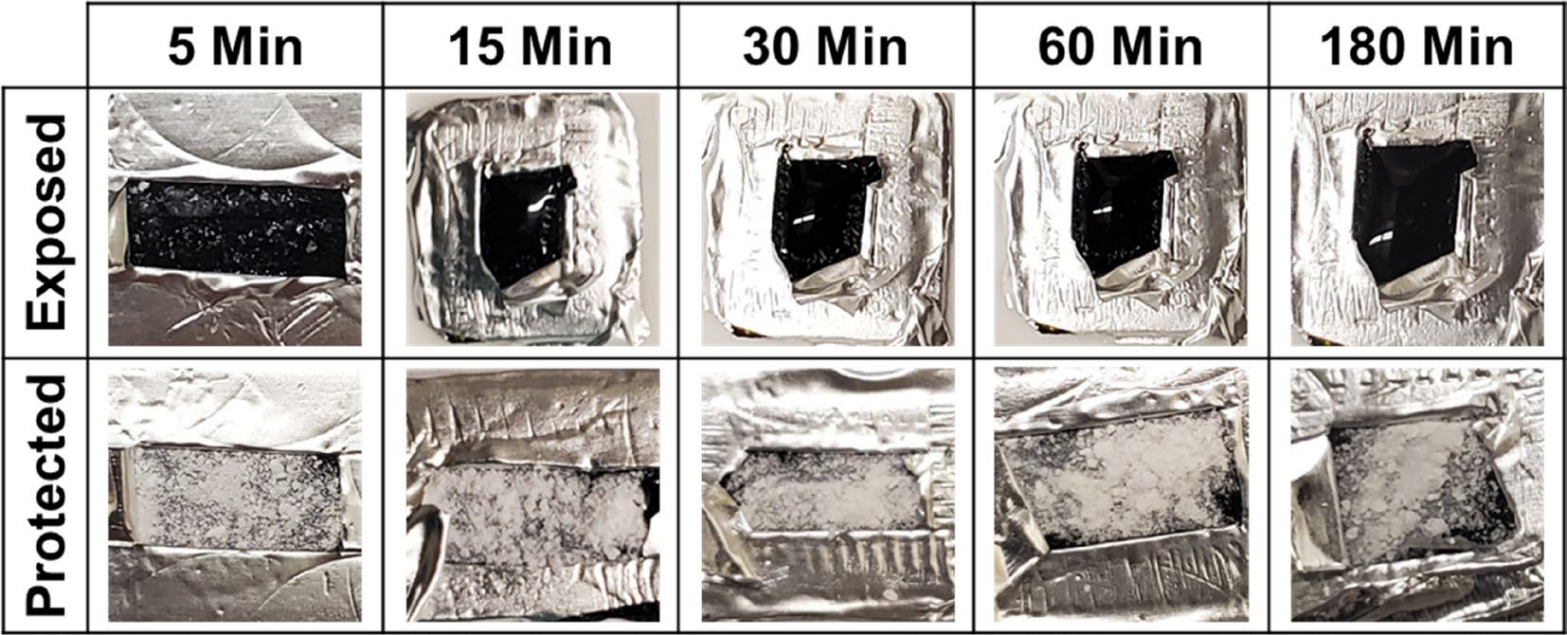
Photos taken of metal stubs placed in the HB for the specified time. The dark appearance of the samples in the “exposed” row is consistent with transitions to a fully hydrated brine. The appearance of the samples in the “protected” row is consistent with no loss in crystalline MgCl_2_, and there is no detectable hydration, even after 180 min in the humidity box (HB).

**FIGURE 4:**
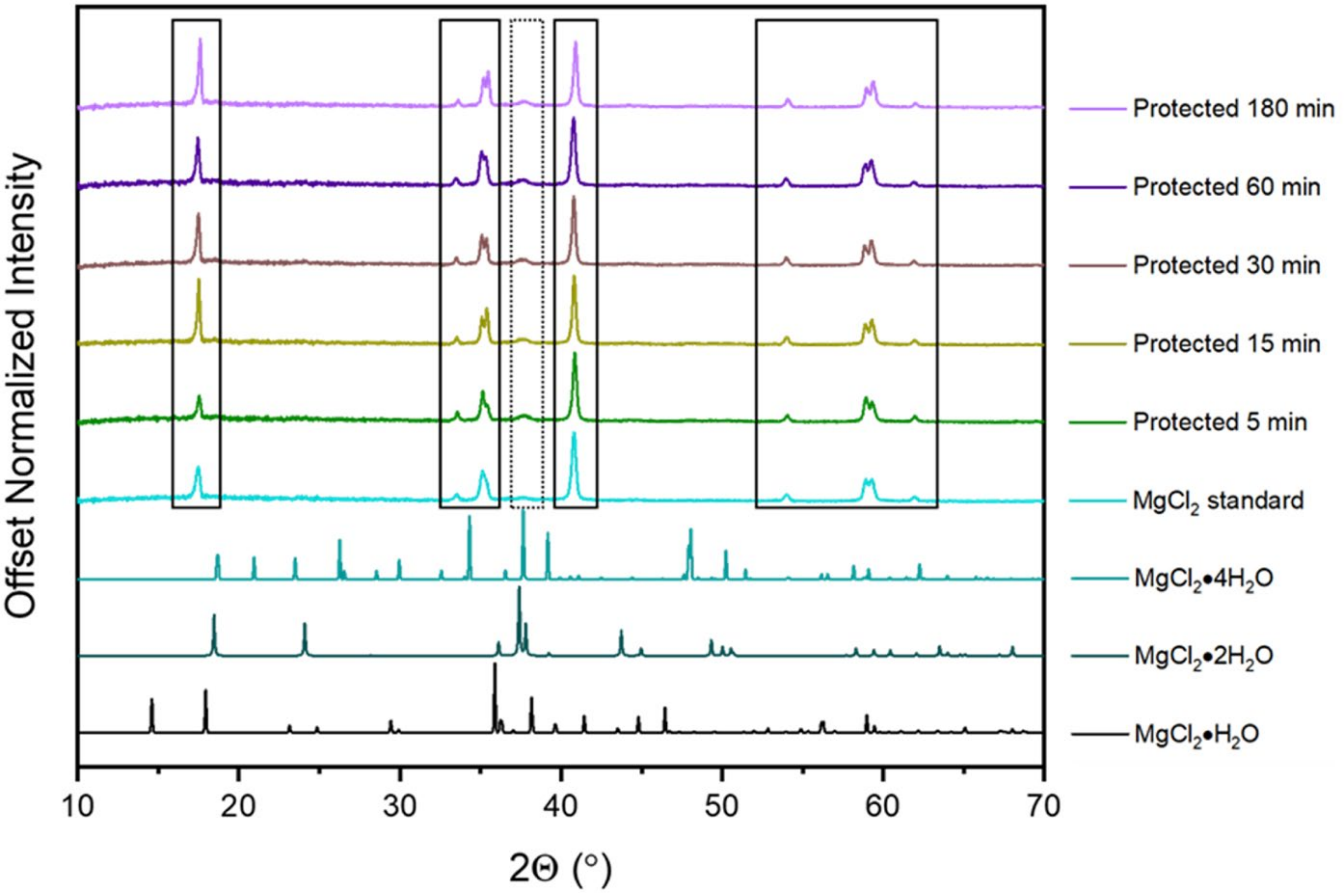
Powder X‐ray diffraction patterns of the protected MgCl_2_ samples exposed to the humidity box (HB), the unexposed MgCl_2_ standard sample, and reference patterns for MgCl_2_, MgCl_2_·H_2_O (Sugimoto et al. 2007), MgCl_2_·2H_2_O (Sugimoto et al. 2007), and MgCl_2_·4H_2_O (Schmidt et al. 2012). All patterns were normalized to the most intense peak and then offset on the *y*‐axis for comparison. The black boxes are representative of anhydrous MgCl_2_ peaks, while the blue box corresponds to a peak found in the pattern of MgCl_2_·4H_2_O.

**FIGURE 5:**
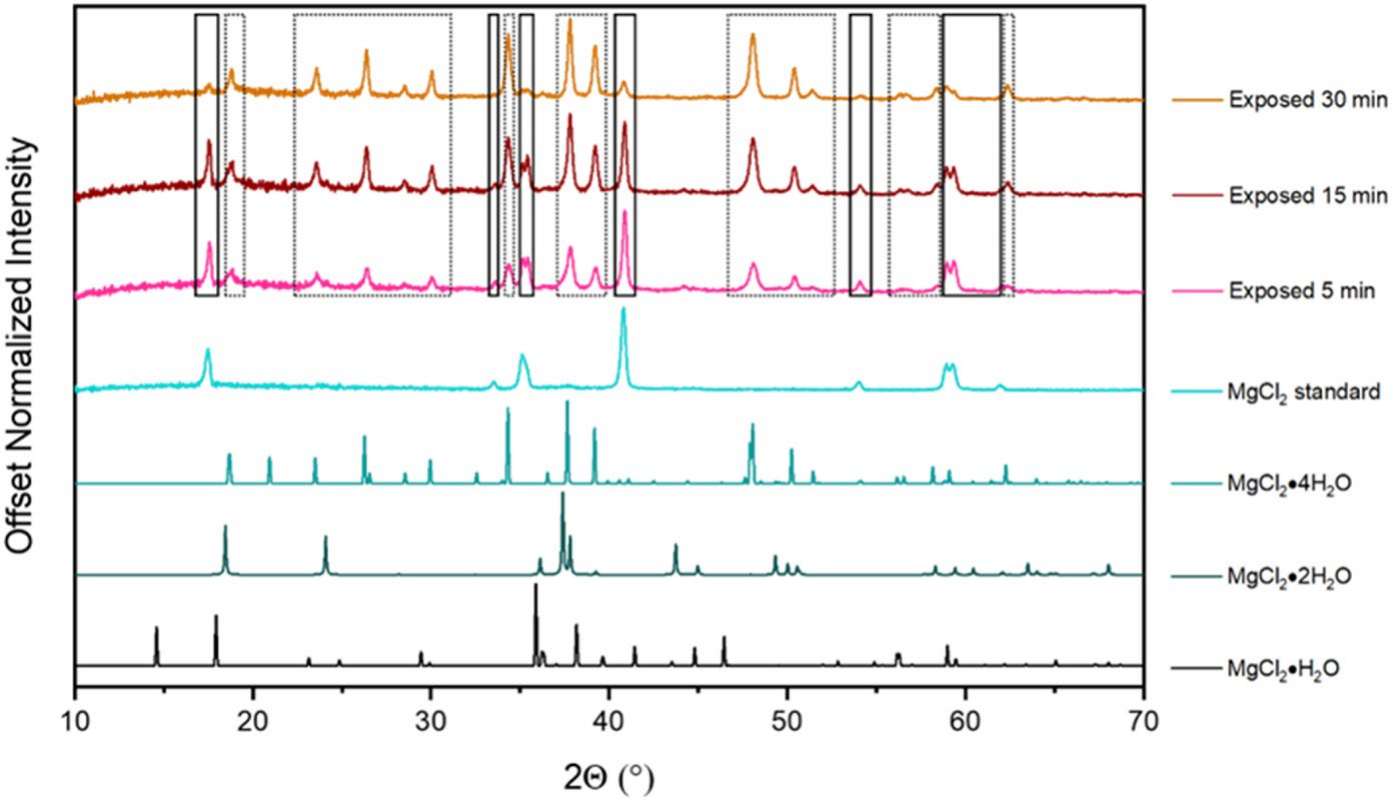
Powder x‐ray diffraction patterns of MgCl_2_ samples exposed to the conditions of the humidity box (HB), the unexposed MgCl_2_ standard sample, and reference patterns for MgCl_2_·H_2_O (Sugimoto et al. 2007), MgCl_2_·2H_2_O (Sugimoto et al. 2007), and MgCl_2_·4H_2_O (Schmidt et al. 2012). All patterns were normalized to the most intense peak and then offset on the y‐axis for comparison. The black boxes correspond to the anhydrous MgCl_2_ standard sample, and the dashed boxes correspond to MgCl_2_·4H_2_O.

**FIGURE 6:**
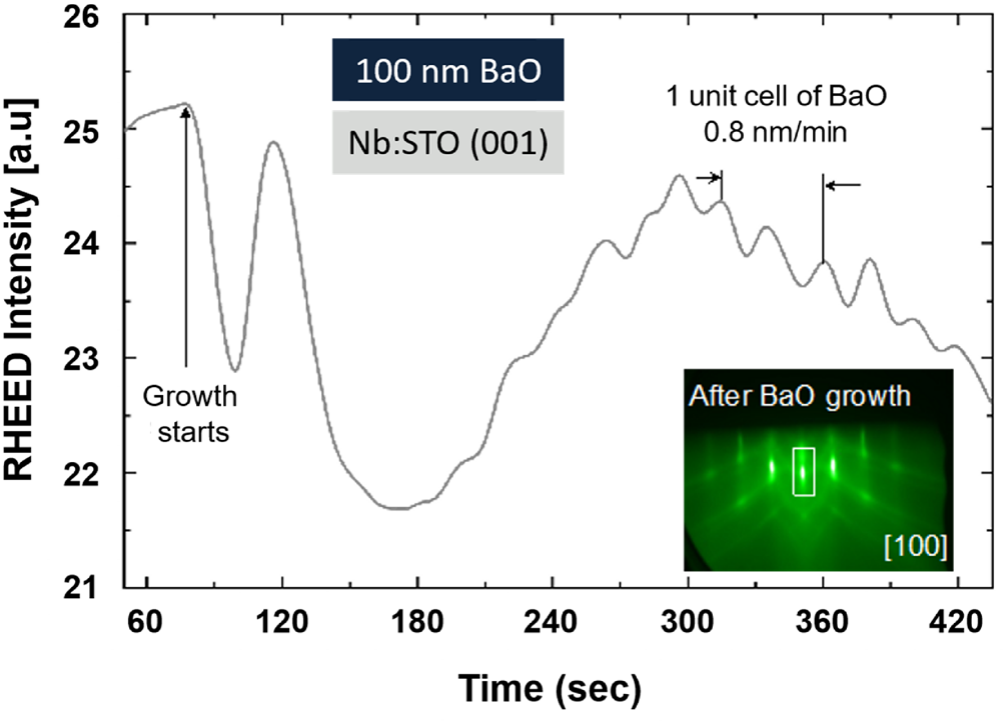
Epitaxial single crystalline BaO thin film on Nb‐doped SrTiO_3_ (STO) (001) substrate. Inset shows the reflection high‐energy electron diffraction (RHEED) image after 100 nm BaO growth along [100]. Note the RHEED intensity oscillations during BaO growth. The period of oscillations determines the growth rate of BaO as 0.8 nm/min.

**FIGURE 7:**
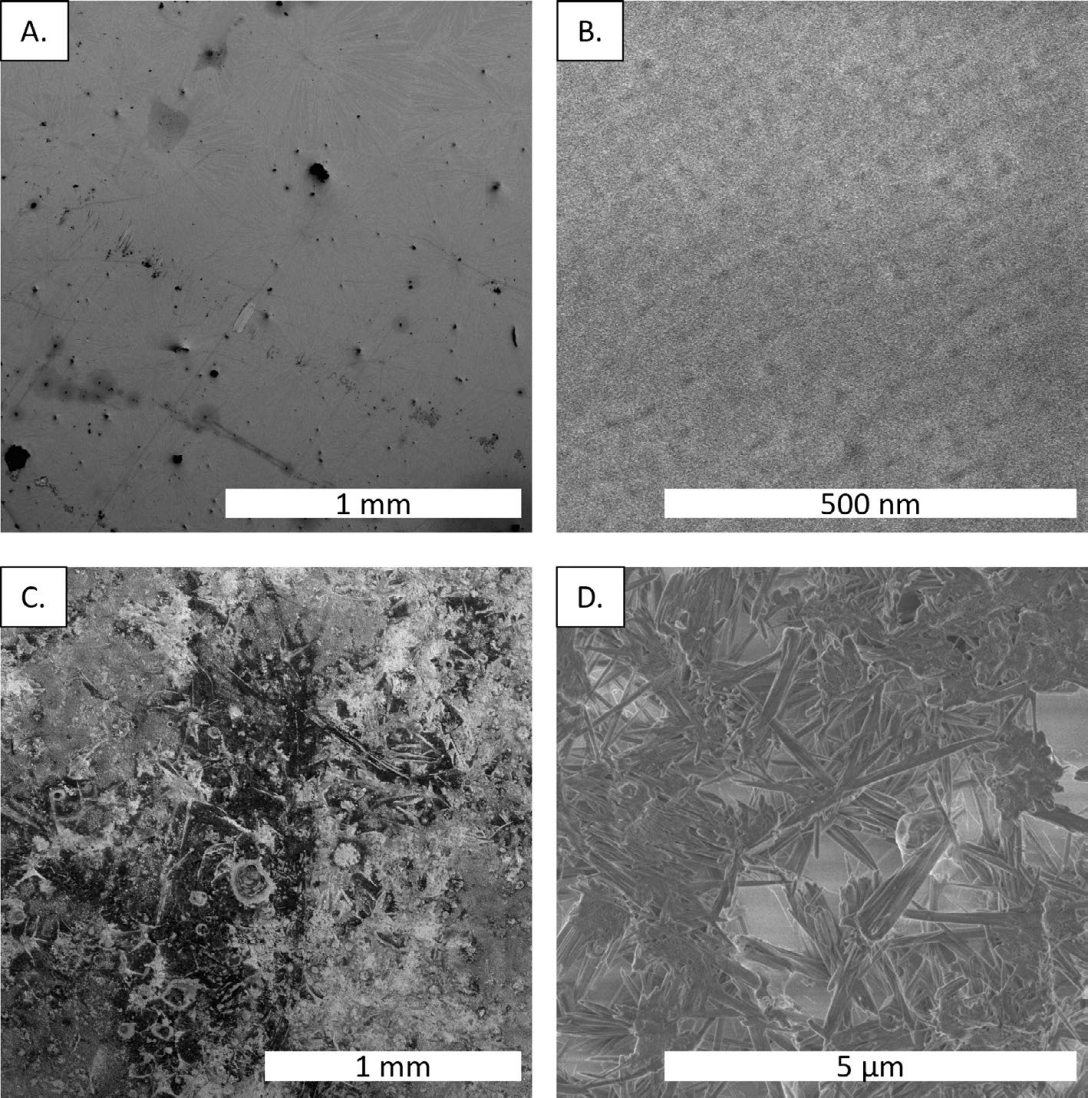
Protected (A, B) and humidity‐exposed (C, D) scanning electron micrographs of the BaO surface. Note that when protected (A, B), BaO exhibits a smooth surface even at incredibly high magnification, whereas the sample that was exposed to 100% humidity for 5 min has noticeable surface roughness (C) and crystal growth (D).

At the SEM station, the plastic container containing the BaO sample was removed from the desiccator and transferred into an in‐house fabricated plexiglass box (Figure 2‐1) that was placed over the sample loading station of a Leica vacuum cryo manipulation system (Leica EM VCM; Figure 2‐2). To purge the box (Figure 2‐1), a continuous flow of N_2_ gas was maintained using an integrated port. The measured relative humidity in the plexiglass box (Figure 2‐1) was 14%. In the box, the sample stub was then removed from the sealed plastic container and mounted onto the Leica cryo‐SEM sample mount as shown in Figure 2‐3. The mounted sample was then transferred into a Leica EM ACE600 high vacuum sputter coater (Figure 2‐4) using a Leica vacuum cryo transfer system (Leica EM VCT500; Figure 2‐5) via the exchange ports on the VCM and ACE600 (Figure 2‐6 and Figure 2‐7, respectively). A knife attached via a sealed port in the ACE600 (Figure 2‐8) was then used to cut open the foil pouch and expose the sample. After opening the foil pouch with the knife, the non‐coated BaO sample was transferred to the SEM (Hitachi SU8230) with the VCT500 for surface characterization. Note that the coated sample, discussed in Supporting Information S1: Section 3, was sputter‐coated with 2 nm of Pt prior to transfer to the SEM; micrographs of this sample can be found in Figure S8. This Hitachi SU8230 microscope is equipped with an energy‐dispersive x‐ray spectroscopy (EDS) detector (Oxford Instruments X‐max 80 N), which was used to perform elemental analysis on the sample (15 kV accelerating voltage, ~15 mm working distance). Finally, standard micrographs were collected at an accelerating voltage of 1 kV, an emission current of 4000–10,000 nA, and a working distance of 5 or 10 mm.

Following characterization, the BaO sample was extracted from the SEM and exposed to 100% relative humidity for 5 min; humidity was controlled with the use of a VITROBOT (ThermoFisher Mark IV system). This exposed sample was then brought back into the SEM to observe sample degradation and collect EDS data.

## 3 Results and Discussion

### 3.1 Method Validation for Smaller Sample Volumes

The aluminum foil pouch protects MgCl_2_ samples prepared as they would be for characterization via SEM. As shown in Figure 3, the exposed CS was nearly completely hydrated after 5 min in the HB and transformed into a brine puddle by the 15‐min time point. Conversely, samples that were in the HB under protective conditions are shown to remain crystalline and white even after 3 h in the HB. The lack of visual changes for the protected salt layer provides sufficient qualitative evidence that the proposed protection method works for scaled‐down sample volumes prepared in a manner suitable for SEM imaging.

### 3.2 Powder XRD

The aluminum foil pouch effectively protects samples from exposure to oxygen or other gases in air, which could cause contamination or degradation of the sample. For the protected samples, no evidence of contamination, additional hydration, or degradation of the MgCl_2_ was detected by XRD (Figure 4), while the XRD patterns of the exposed samples were consistent with MgCl_2_ hydrates (Figure 5). The pattern of the control (see Figures 4 and 5; MgCl_2_ standard) is consistent with anhydrous MgCl_2_ with the addition of a small, broad peak at ~37.7° 2*θ*, likely corresponding to MgCl_2_·4H_2_O that may have developed upon exposure to the atmosphere when the sample was extracted from the vacuum oven (Figure S4). While the intensity of the ~37.7° 2*θ* peak (Figure 4) appears increased for the protected sample set relative to the same peak for the control sample, this can likely be attributed to the short exposure to ambient conditions during the transfer of each sample from the protected glass slide into a vial and subsequently back into the glovebox. This exposure to ambient conditions did not occur with the control sample. Overall, the control and protected samples are primarily anhydrous MgCl_2_ with a trace amount of either MgCl_2_·4H_2_O or MgCl_2_·2H_2_O detected by XRD. In contrast, samples exposed to the HB environment without the protective foil envelope are mixtures of the two phases, with the fraction of MgCl_2_·4H_2_O increasing with increased exposure time (Figure 5). Note that the exposed sample at the 60‐ and 180‐min time points is absent from Figure 5; the 180‐min sample became a liquefied brine mixture while the 60‐min sample had peaks assigned to anhydrous MgCl_2_, MgCl_2_·4H_2_O, MgCl_2_·8H_2_O, and MgCl_2_·12H_2_O (Figure S5).

### 3.3 BaO Crystal Growth and SEM Characterization

As shown in the inset of Figure 6, after the growth of BaO, a streaky reflection high‐energy electron diffraction (RHEED) pattern with clear Kikuchi lines is obtained, suggesting epitaxial single crystalline growth and a smooth surface morphology. As a result, we anticipated that the protected BaO samples would exhibit a smooth surface morphology when characterized via SEM, as is observed in Figure 7A,B. It is worth noting that the dark regions present in Figure 7A are likely representative of BaO hydration to Ba(OH)_2_ (Varshney et al. 2024). However, this is an insignificant amount of hydration, as evidenced by the bulk EDS data (Figure S7), which indicates a nearly pure BaO material (Ba:O experimental weight ratio of 10.34 compared to a theoretically pure BaO weight ratio of 8.58 Ba:O). After exposure to 100% humidity for 5 min, the material exhibits a roughened surface (Figure 7C,D), and the resultant EDS of the bulk surface indicates full hydration of the material to Ba(OH)_2_ (Figure S7; experimental Ba:O weight ratio of 5.04 compared to a theoretical weight ratio of 4.29). As a result of this significant transformation after exposure to ambient humidity, we believe there is sufficient evidence demonstrating the effectiveness of the outlined sample protection method for this highly hygroscopic material. Finally, the SEM micrograph for the sputter‐coated BaO sample can be seen in Figure S8 and demonstrates the ability to (1) seamlessly sputter coat samples with the preparation method outlined herein and (2) detect defects in the sample after exposure to humidity.

### 3.4 Aluminum and Carbon Tape Permeability

Since MgCl_2_ and BaO do not readily react with ambient nitrogen or oxygen like Li or SrI_2_, the data presented above cannot be used directly as a proof‐of‐concept test to demonstrate this method's effectiveness against protecting from these potential contaminants. However, it is still worth discussing this method in relation to preventing nitrogen and oxygen exposure. Johansson et al. (2021) have reported that an 11‐μm‐thick aluminum foil with even one pinhole (diameter = 0.3 mm) can completely compromise the oxygen barrier, and aluminum foil is susceptible to pinhole formation and tears due to its ductility (Kerry 2012; Dunn 2015). However, pinhole formation is also known to cause significant loss in water vapor protection (Johansson et al. 2021), which is not evident based on the data collected from this study. Further, the level of protection from both ambient oxygen and water can be easily increased since heavier gauges of aluminum foil (> 17 μm) (Kerry 2012) provide a complete barrier to gases and liquids (compared to the water vapor transfer rate (WVTR) for an 11 μm thick foil with no pinholes of < 0.001 g/m^2^ × 24 h at a temperature of 27°C and RH of 50% (Johansson et al. 2021), WVTR for a 9‐μm‐thick foil of 0.3 g/m^2^ × 24 h at 38°C and RH of 90% (Kerry 2012), and oxygen transfer rate (OTR) of an 11‐μm‐thick foil of 0.05 cm^3^/m^2^ × 24 h) (Johansson et al. 2021) and the use of various coatings or the incorporation of the foil into laminates can increase strength and/or counteract the weaknesses of pure foils (Johansson et al. 2021; Kerry 2012). Also, when comparing the WVTR and OTR of pinhole‐free aluminum foil to the corresponding values of various elastomeric materials (Gaume and Joubert 2011), both WVTR and OTR are orders of magnitude lower for the foil, demonstrating advantages of this protection method for atmosphere‐sensitive materials.

The carbon tape is another component of this method that could allow for the exposure of the sample to ambient conditions. The WVTR and OTR are unknown for this material, though given the associated low values for pristine aluminum foil, it is likely the weakest link in the preparation method. Unlike the foil, which is only (on the order of) 10 μm thick, the width of material that a gas and/or liquid would need to permeate through the tape is on the order of millimeters (the adhered surface of the foil to the tape) on all edges depending on the method—at least 1 mm for the metal stubs and 3 mm for the glass slides. As such, we suspect that the tape would provide enough of a barrier with the transfer method detailed above that it would be sufficient. Alternatively, the method could be adjusted by trimming the edge of the carbon tape such that it is not flush with the edges of the metal stub. Epoxy could then be applied to these exposed metal surfaces, and the foil could be affixed to it. This may require the use of a larger metal stub, but if applied correctly, it would provide a guaranteed hermetic seal between the metal stub and foil once cured.

## 4 Conclusion

Here, we have demonstrated that the proposed sample preparation method effectively protects the atmosphere‐sensitive materials MgCl_2_ and BaO from exposure to atmospheric contaminants. MgCl_2_ samples that were prepared on the metal stubs, protected by the aluminum foil pouch, and exposed to the HB remained crystalline and white after 3 h of exposure to high humidity conditions compared to an unprotected sample that turned into a brine puddle after just 15 min of exposure. Likewise, XRD patterns suggest that the MgCl_2_ samples that were prepared on glass slides and protected with aluminum foil remained as pure MgCl_2_ after extended exposure to the HB, while unprotected bulk MgCl_2_ transformed into various MgCl_2_ hydrates under identical conditions. The effectiveness of the protection method was also confirmed with mass‐gain experiments, which demonstrated that the protected MgCl_2_ had no appreciable mass gain (due to water uptake) after even 6 h of exposure to high humidity. In addition, the SEM micrographs confirm the effectiveness of the protection method for the highly hygroscopic BaO material. When protected with the foil pouch and characterized via SEM, the BaO surface remained smooth (as predicted by RHEED), and the EDS data suggested the crystal structure of the material remained as BaO. Following 5 min of exposure to a 100% humid environment, the surface topography changed significantly, and the resultant EDS spectrum suggested that the BaO transitioned to Ba(OH)_2_. When the aluminum foil pouch is used in combination with the Leica Cryo‐EM instrumentation, the complete system provides a preparation option that addresses some of the concerns associated with other common preparation methods for hygroscopic materials (e.g., no sample exposure, materials and instrumentation are readily or commercially available) and can be utilized to characterize a variety of different materials.

Finally, it is worth noting that while air‐lock systems have been used for characterizing atmosphere‐sensitive materials, the versatility demonstrated by the Leica Cryo‐EM instrumentation—designed specifically for cryo‐SEM applications—is another significant benefit. The barrier to entry for the instrumentation is reduced since it provides a solution to characterizing a plethora of diverse materials, and the cost can thus be spread out across research groups with various interests.

## Author Contributions

**Louis G. Corcoran:** conceptualization, investigation, writing – original draft, methodology, validation, writing – review and editing, formal analysis, data curation, visualization. **Ellen M. Monzo:** investigation, writing – original draft, methodology, validation, writing – review and editing, formal analysis, data curation, visualization. **Chinomso E. Onuoha:** investigation, writing – original draft, methodology, validation, writing – review and editing. **Shivasheesh Varshney:** writing – original draft, writing – review and editing, methodology, validation, formal analysis. **Han Seung Lee:** conceptualization, investigation, writing – review and editing, methodology, validation. **Chris Frethem:** conceptualization, writing – review and editing, methodology. **Bharat Jalan:** funding acquisition, writing – review and editing, supervision. **Alon V. McCormick:** funding acquisition, writing – review and editing, supervision. **R. Lee Penn:** funding acquisition, writing – review and editing, supervision.

## Funding

This study was supported by National Science Foundation (DMR‐2011401, DMR‐1229263, and ECCS‐2025124), U.S. Department of Energy (DE‐SC0020211, DE‐SC0023464, DE‐AR0000804, and DE‐EE0007888), Office of the Vice President for Research, University of Minnesota, University of Minnesota Industry Partnership for Research in Interfacial and Materials Engineering (IPRIME), and University of Minnesota West Central Research and Outreach Center.

## Conflicts of Interest

The authors declare no conflicts of interest.

## Supporting information


**Data S1:** Supporting Information.

## Data Availability

Data for this manuscript is publicly available through the Data Repository for the University of Minnesota (DRUM) and can be found with the following identifiable link: https://doi.org/10.13020/0s3h‐wm31.
