# Supplementary material for: Electron Microscopy Transfer System to Protect Atmosphere‐Sensitive Materials for Scanning Electron Microscopy Characterization
Source: Microsc Res Tech. 2025 Dec 31;89(5):759–68. doi: 10.1002/jemt.70107 (PMC13048188; doi:10.1002/jemt.70107)
Supplement: Supplementary file 1 — Data S1: Supporting Information. [file JEMT-89-759-s001.docx]

**Supporting Information for:**

**Electron Microscopy Transfer System to Protect Atmosphere-sensitive Materials for Scanning Electron Microscopy Characterization**

**Authors:** Louis G. Corcoran, Ellen M. Monzo, Chinomso E. Onuoha, Shivasheesh Varshney, Han Seung Lee, Chris Frethem, Bharat Jalan, Alon V. McCormick, and R. Lee Penn

**Supporting Information Section 1: X-ray Diffraction Diffractograms of MgCl_2_ Samples and Standards and Sample Preparation Schematics/Flowcharts**

| **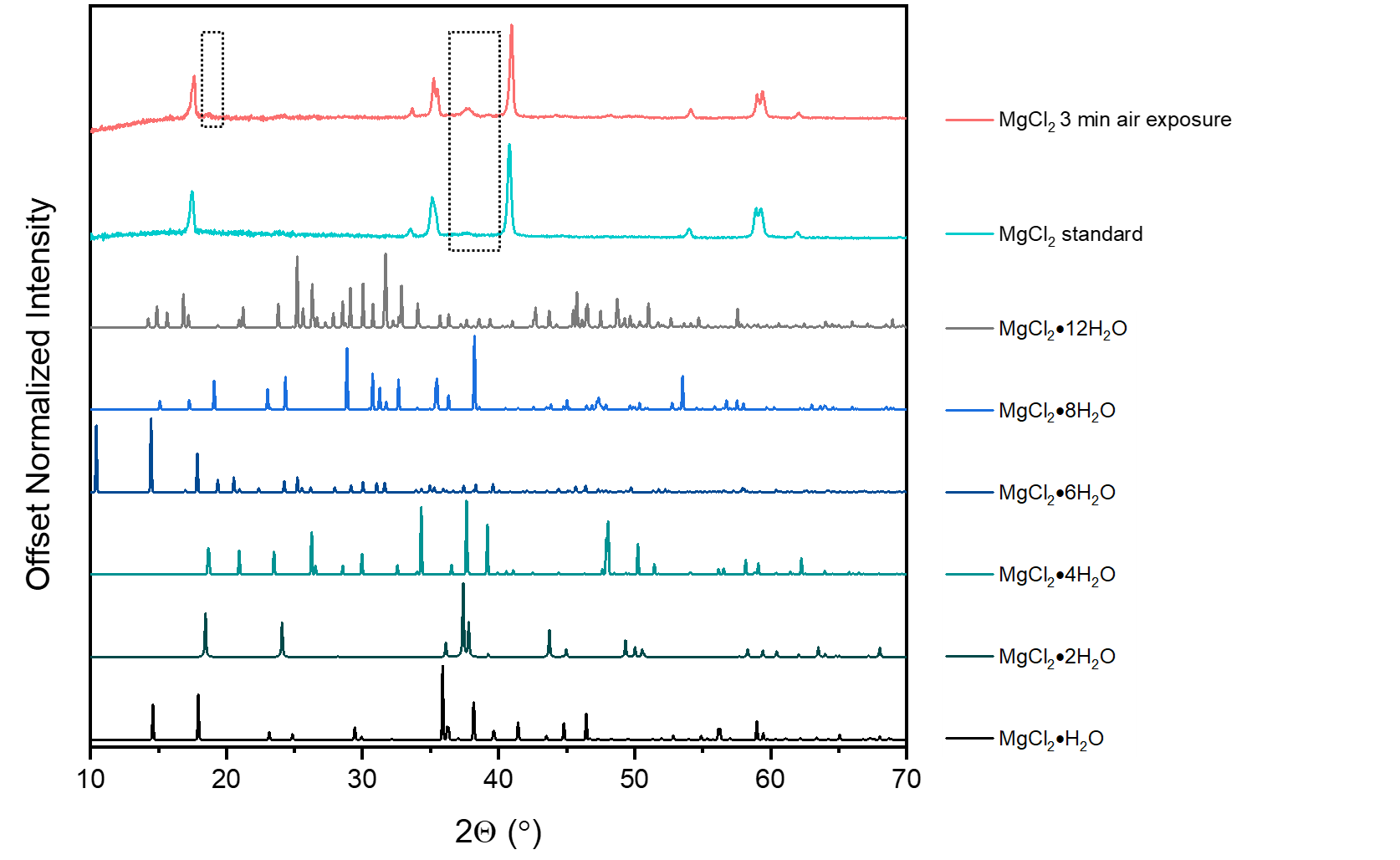.** |
| --- |
| **Figure S1.** Powder X-ray diffraction pattern of anhydrous MgCl_2_ exposed to air for 3 minutes in a humid environment, the unexposed MgCl_2_ standard sample, and reference patterns for MgCl_2_·H_2_O (Sugimoto et al. 2007), MgCl_2_·2H_2_O (Sugimoto et al. 2007), MgCl_2_·4H_2_O (Schmidt et al. 2012), MgCl_2_·6H_2_O (Villars and Cenzual 2016), MgCl_2_·8H_2_O (Hennings et al. 2013), and MgCl_2_·12H_2_O (Hennings et al. 2013). All patterns were normalized to the most intense peak and then offset on the y-axis for comparison. The dashed boxes correspond to MgCl_2_•4H_2_O or MgCl_2_•2H_2_O. |

**Discussion for Figure S1:**

The relative intensity of the peak at 37.7° 2θ increases and a peak at 18.7° 2θ appears in the 3-minute air exposed sample that correspond to either MgCl_2_·4H_2_O or MgCl_2_•2H_2_O.

| 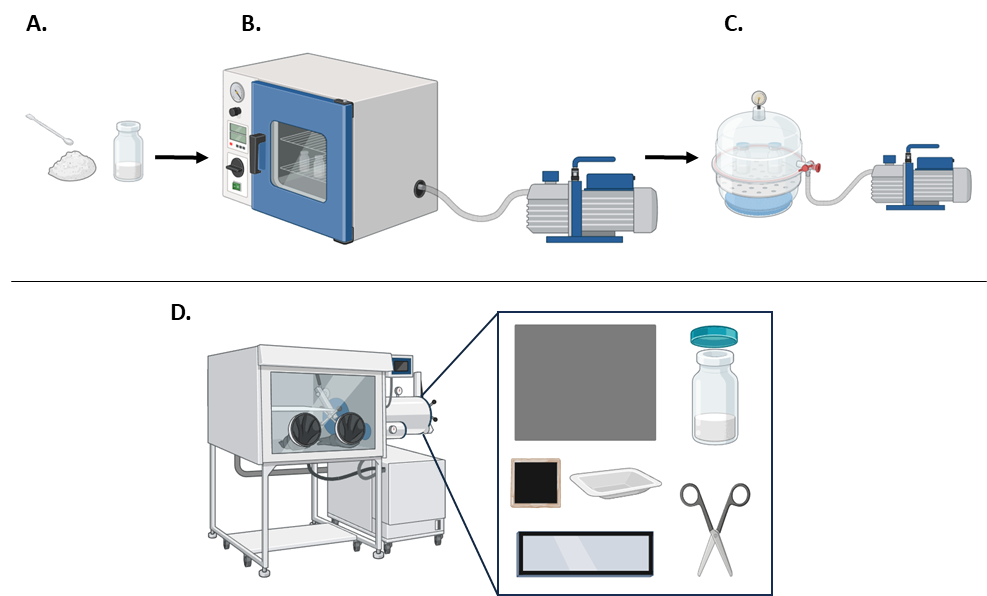 |
| --- |
| **Scheme S1.** Sample preparation schematic for Section 2.1.1 and Section 2.1.2 of the main text. Section 2.1.1: A) Transfer ~5 g of MgCl_2_ to a 20 mL scintillation vial and cover the opening of the vial with aluminum foil. B) Place the salt-containing scintillation vial in a vacuum oven to purify/dry the MgCl_2_; the temperature, pressure, and dry time are outlined in section 2.1.1 of the main text. C) Remove the vial(s) from the oven and immediately cap and wrap with parafilm. Store the vial(s) of dry MgCl_2_ in a vacuum desiccator. Section 2.1.2: Remove a MgCl_2_ vial from the desiccator and remove the cap. Immediately transfer the vial and cap, along with aluminum foil, plastic weigh boats, glass slides, carbon tape, and a pair of scissors into the antechamber of a glovebox. Close the antechamber and cycle the chamber between vacuum and N_2_ gas multiple times before transferring all components into the glovebox. This figure was partially created in BioRender.com. |

| 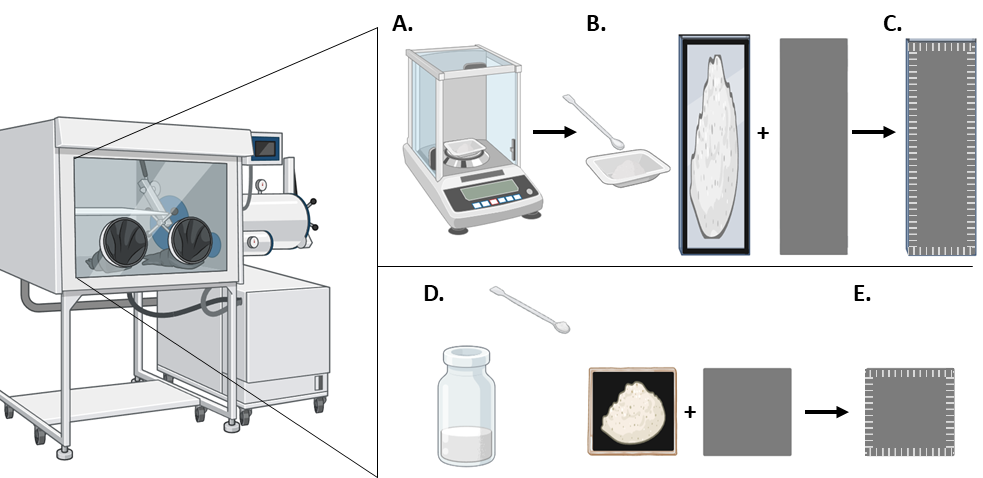 |
| --- |
| **Scheme S2.** Sample preparation schematic for Section 2.1.3 and Section 2.1.4 of the main text. Section 2.1.3: A) utilizing the materials brought into the box in Figure S1.D, deliver 0.15 g of MgCl_2_ to 10 weigh boats. B) Transfer the MgCl_2_ from five different weight boats to five different glass slides and spread the salt evenly across the slide with a spatula. Cut five pieces of aluminum foil with, dimensions slightly larger than each of the glass slides, and set aside. C) Apply the pieces of aluminum foil to the carbon tape (black outline on the slide in S2.B) on each prepared slide and press the foil firmly and evenly across the tape edges surrounding the sample with the use of a tweezers or rounded spatula. Note that no additional steps need to be taken for the five remaining weigh boats containing MgCl_2_. Section 2.1.4: D) utilizing the materials in Figure S1.D apply a qualitative amount of MgCl_2_ to the small metal stub (carbon tape pre-affixed) such that each has ~75% of the tape covered. Cut pieces of aluminum foil with dimensions slightly larger than each of the glass slides, and set aside. E) Apply the pieces of aluminum foil to the carbon tape on each prepared stub and crimp the foil to the exposed tape edges with a metal tweezer or spatula. This figure was partially created in BioRender.com. |

| 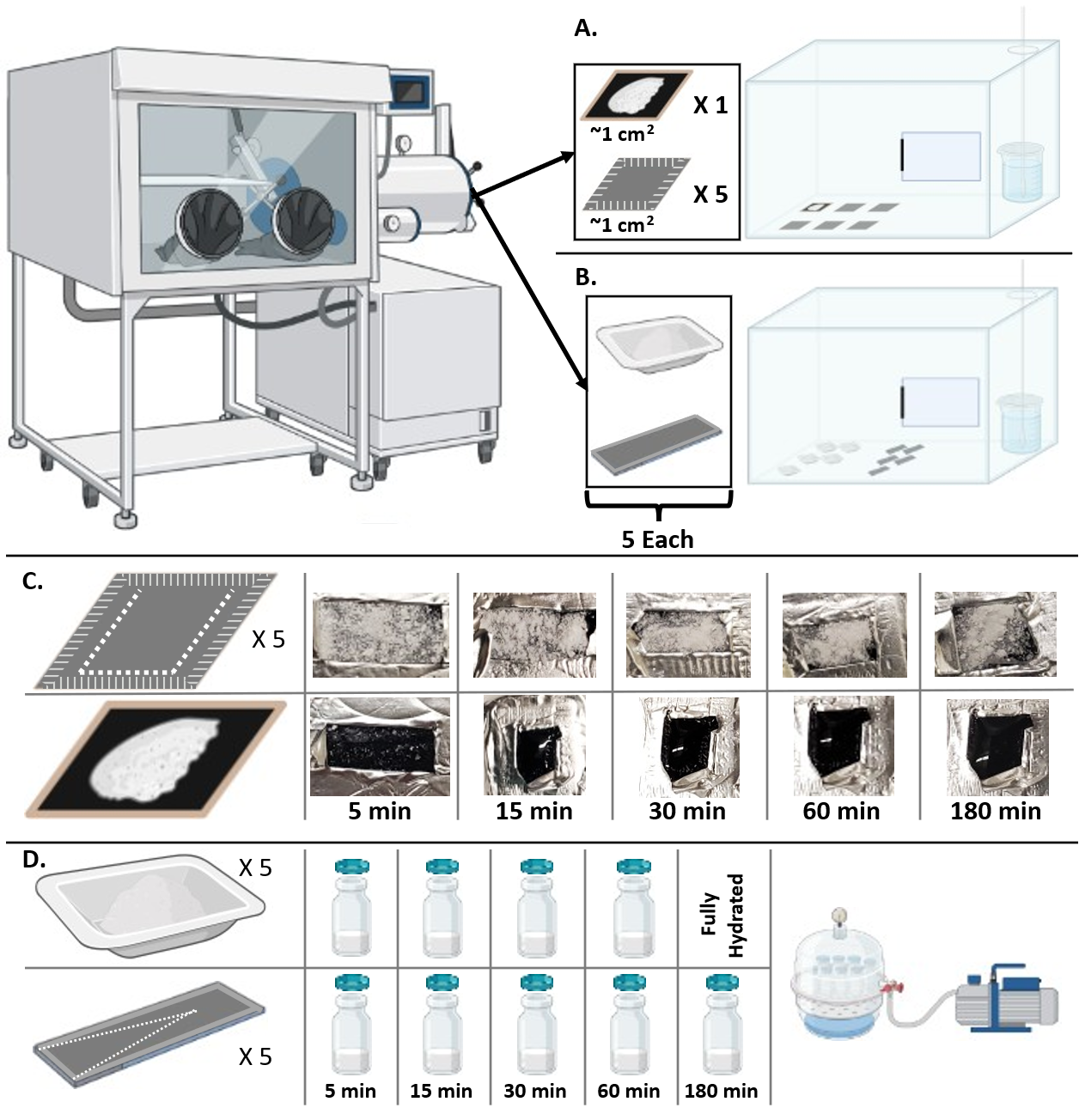. |
| --- |
| **Scheme S3.** Sample preparation schematic for Section 2.1.5 in the main text; (A,C) correspond to the metal stubs while (B,D) to the glass slide/weigh boats; the glovebox preparation for each is included in Scheme S2. Note that the clear box in both (A) and (B) is representative of a plexiglass box that incorporated a gasketed door on the front and a port in the top right corner that opened above a water filled beaker with a gas dispersion tube going through the port into the water. Air was circulated through this tube to increase the humidity of the box to 90%+ relative humidity. A) Remove six prepared metal stubs from the glovebox and transfer them into the humidity box; remove the foil from one stub (or prepare without foil) prior to transferring into the humidity box. C) At each designated timepoint: remove the exposed stub and take a photo of the stub before returning it to the humidity box. Remove one of the foil-protected stubs and cut the foil open along the dashed line to expose the salt – take a photo immediately after the MgCl_2_ is exposed. B) Remove five weigh boats and 5 glass slides (with protected MgCl_2_) from glovebox and immediately transfer into the humidity box. D) At each timepoint, transfer the MgCl_2_ material from (i) one weigh boat and (ii) one foil-protected slide (cut along the dotted line to expose the MgCl_2_) into a dried scintillation vial and cap/parafilm immediately. Store the samples in a vacuum desiccator for later use. This figure was partially created in BioRender.com. |

| 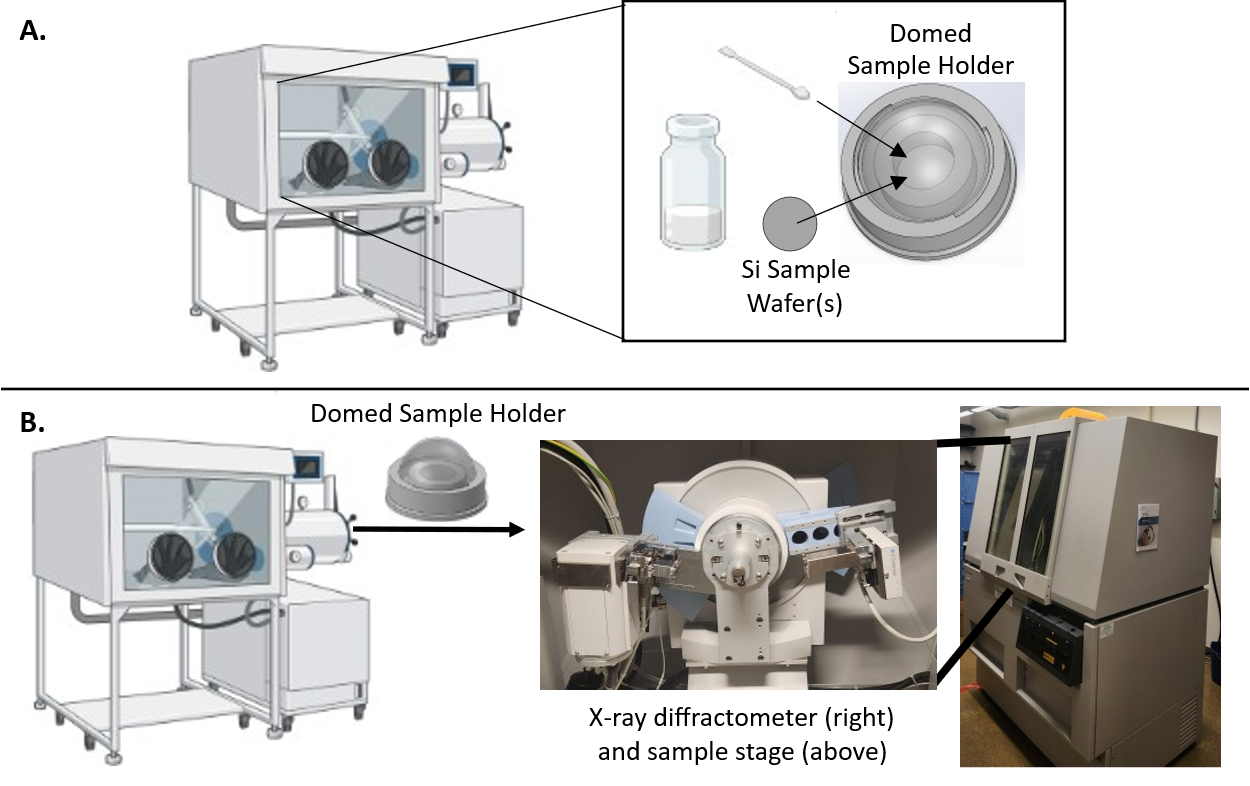 |
| --- |
| **Scheme S4.** Sample preparation schematic for Section 2.1.6 in the main text. Remove the salt sample of interest from the vacuum desiccator (Scheme S3.D). Remove the cap and place the vial and cap, along with the Anton Paar domed sample holder components (for XRD characterization) and zero background Si sample wafers into the glovebox antechamber and immediately cycle the materials into the glovebox. A) Place the Si sample wafers into the sample cavity of the domed sample holder. (Note that enough Si sample wafers should be stacked into the sample cavity such that the remaining space can be filled) with a thin layer of sample.) Following this, load the sample cavity with sample, taking care not to overfill the cavity, and affix the plastic dome to the sample stage with the provided tool in the Anton Paar kit. B) Immediately remove the sample from the glovebox and load it into the XRD for the diffraction measurement. The program utilized for this work is outlined in the main text (Section 2.1.6). This figure was partially created in BioRender.com. |

| **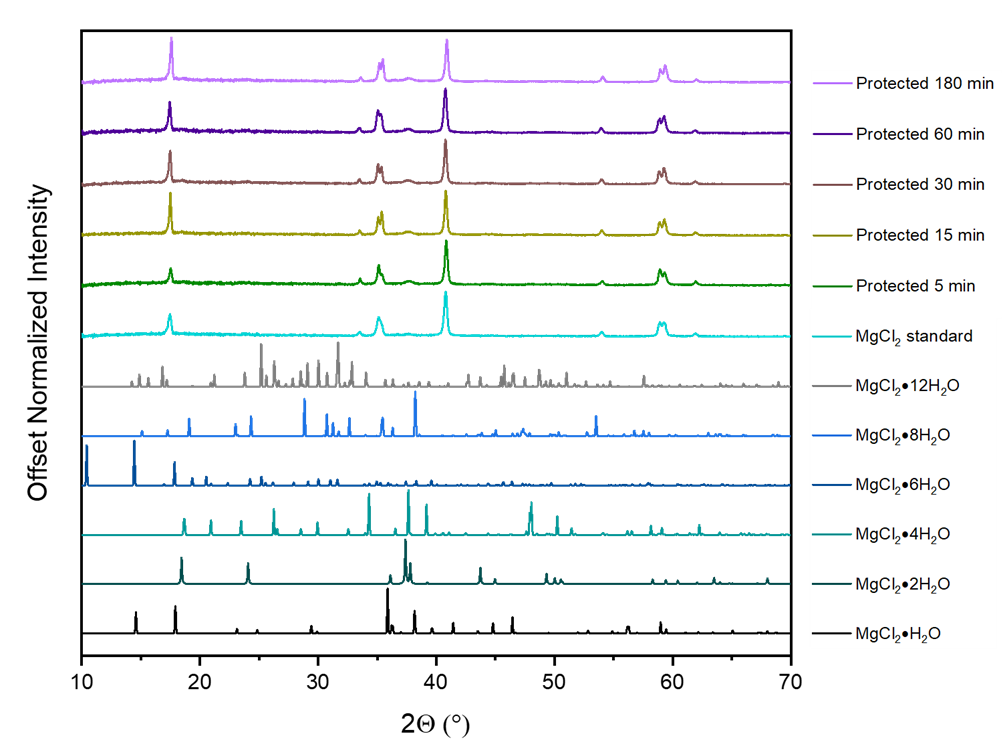.** |
| --- |
| **Figure S2.** Powder X-ray diffraction patterns of the protected MgCl_2_ samples exposed to the HB, the unexposed MgCl_2_ standard sample, and reference patterns for MgCl_2_·H_2_O (Sugimoto et al. 2007), MgCl_2_·2H_2_O (Sugimoto et al. 2007), MgCl_2_·4H_2_O (Schmidt et al. 2012), MgCl_2_·6H_2_O (Villars and Cenzual 2016), MgCl_2_·8H_2_O (Hennings et al. 2013), and MgCl_2_·12H_2_O (Hennings et al. 2013). All patterns were normalized to the most intense peak and then offset on the y-axis for comparison. |

| .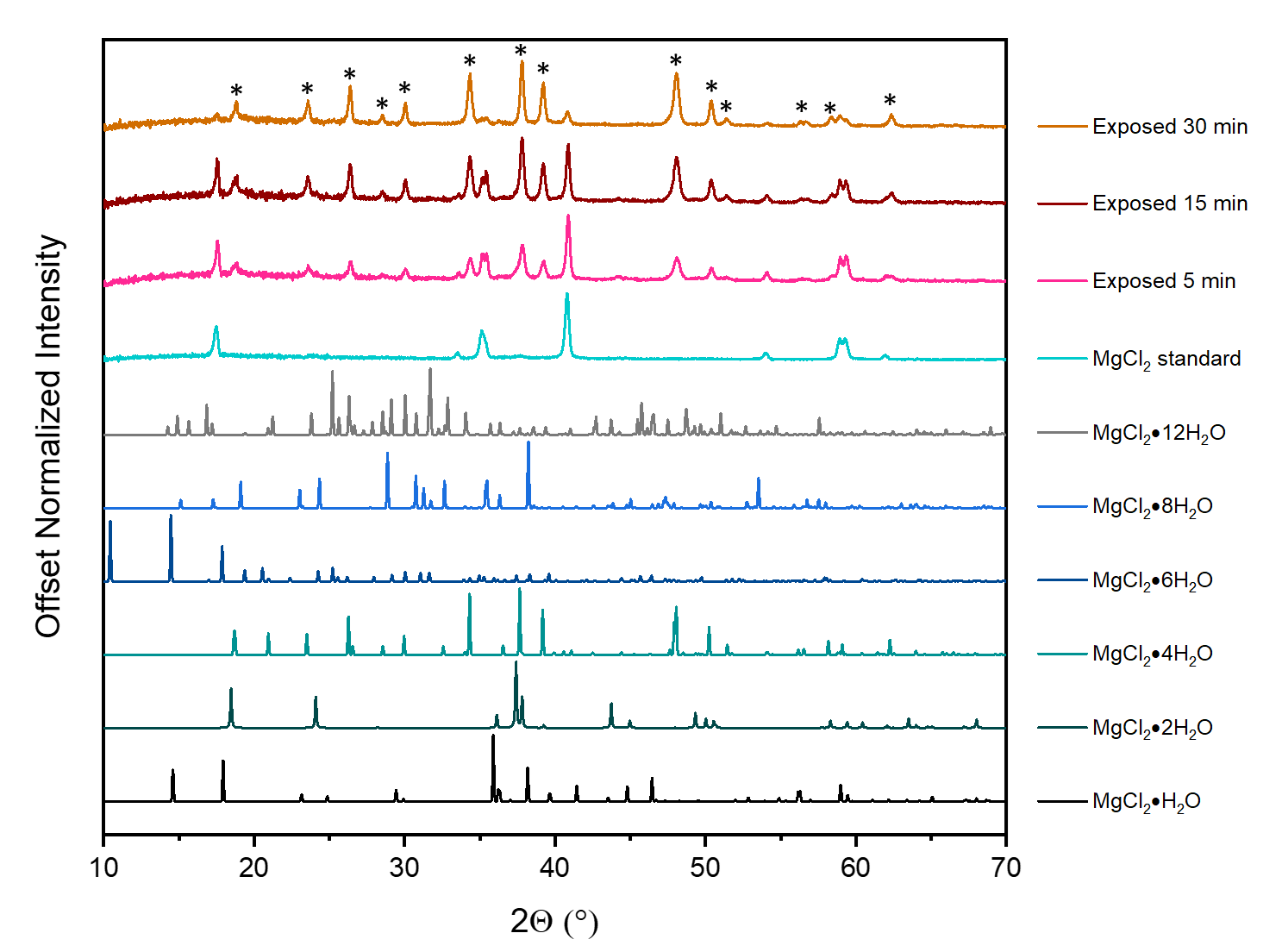 |
| --- |
| **Figure S3.** Powder X-ray diffraction patterns of MgCl_2_ samples exposed to the conditions of the HB, the unexposed MgCl_2_ standard sample, and reference patterns for MgCl_2_·H_2_O (Sugimoto et al. 2007), MgCl_2_·2H_2_O (Sugimoto et al. 2007), MgCl_2_·4H_2_O (Schmidt et al. 2012), MgCl_2_·6H_2_O (Villars and Cenzual 2016), MgCl_2_·8H_2_O (Hennings et al. 2013), and MgCl_2_·12H_2_O (Hennings et al. 2013). Asterisk symbols have been added to the topmost pattern to more clearly denote the presence of hydrated phases. All patterns were normalized to the most intense peak and then offset on the y-axis for comparison. |

| 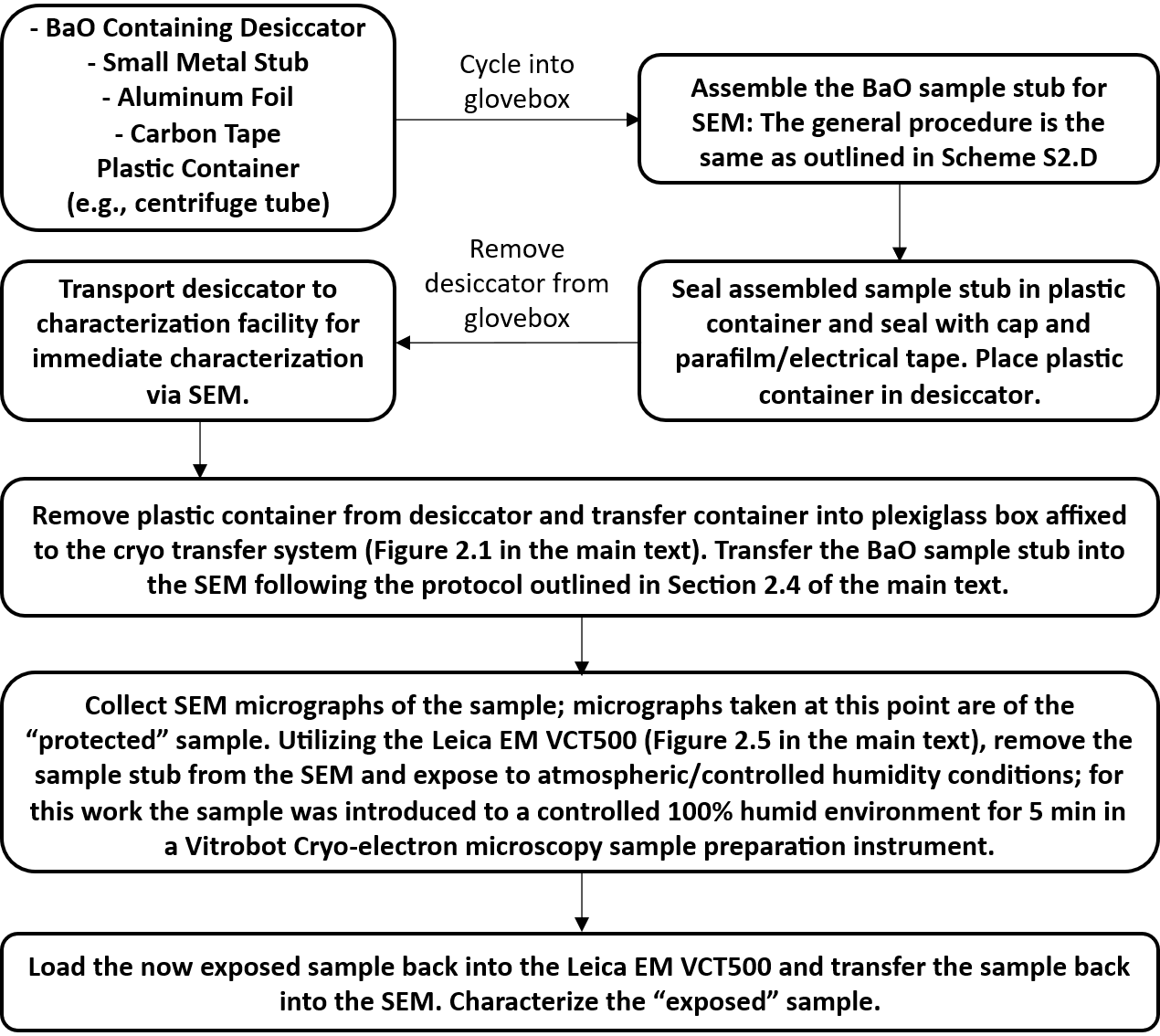 |
| --- |
| **Scheme S5.** Flowchart corresponding to section 2.4 of the main text outlining the workflow for sample preparation and subsequent scanning electron microscopy characterization of the BaO protected sample. The chart covers the entire workflow beginning with materials being brought into the N_2_ glovebox for “protected” sample preparation through characterization of the “exposed” sample. A more detailed description of the Leica EM cryo transfer system and its integration within the workflow is outlined in Figure 2.1 of the main text. |

| 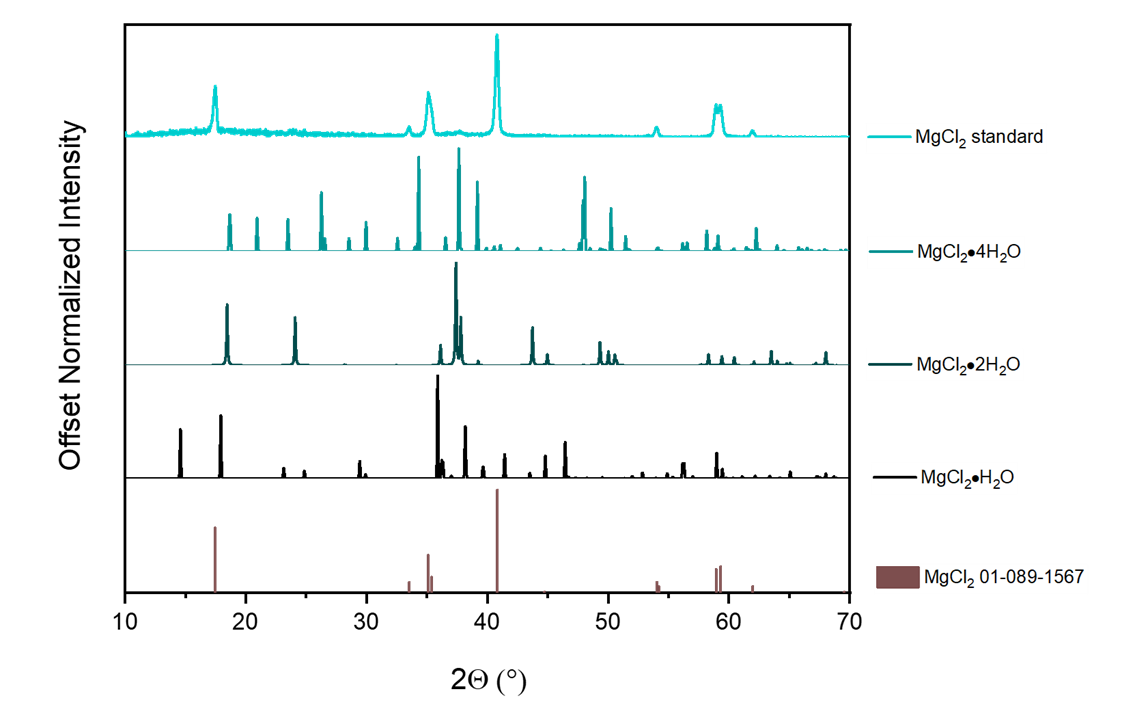. | |
| --- | --- |
| **Figure S4.** Powder X-ray diffraction pattern of the MgCl_2_ standard control sample compared to reference patterns for MgCl_2_ (PDF 01-089-1567), MgCl_2_·H_2_O (Sugimoto et al. 2007), MgCl_2_·2H_2_O (Sugimoto et al. 2007), and MgCl_2_·4H_2_O (Schmidt et al. 2012). All patterns were normalized to the most intense peak and then offset on the y-axis for comparison. |  |

| 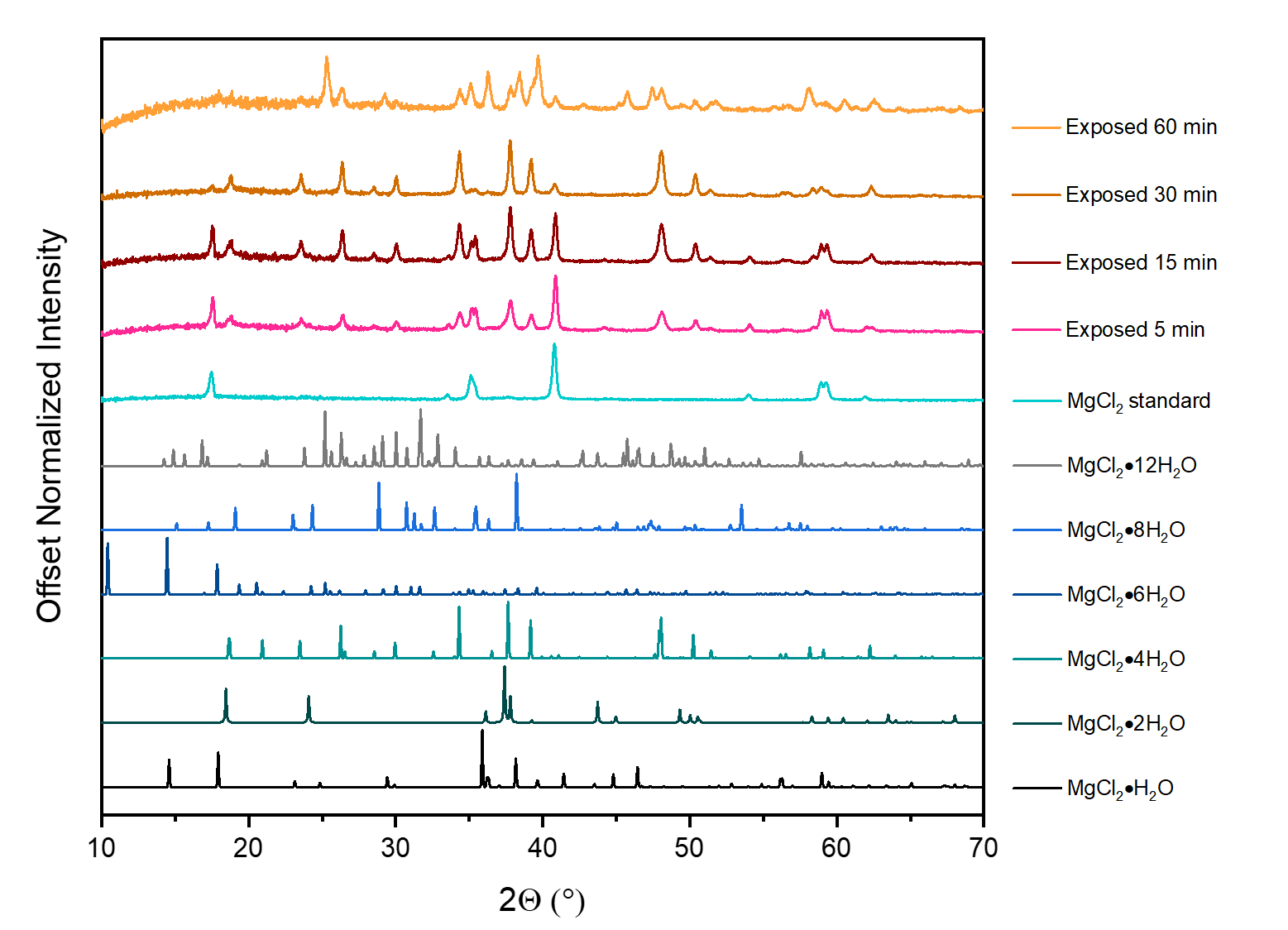 |
| --- |
| **Figure S5.** Powder X-ray diffraction patterns for all exposed MgCl_2_ samples (up to 60 minutes of exposure) along with reference patterns (Hennings et al. 2013; Schmidt et al. 2012; Sugimoto et al. 2007; Villars and Cenzual K 2016). |

**Supporting Information Section 2: Mass-gain Experiments**

*Experimental Procedure:*

Mass-gain experiments were conducted to determine if any water permeated the protected sample enclosure but may not have resulted in a change in the crystal structure of bulk MgCl_2_. Four samples were prepared in the same manner as described in section 2.1.3 and the mass of the MgCl_2_ applied to each slide was recorded. After each slide was fully prepared (i.e. Figure 1-A3) they were labeled (1-4) and massed six times on an analytical balance, three measurements were taken inside the glovebox and three outside of the glovebox (the average masses of these measurements are in Table 1; Mettler Toledo); this was done to ensure the precision and accuracy of the two balances. The mass of salt on each slide was then subtracted from the total mass of the corresponding slide to determine the mass of the apparatus without salt (*M*_aws_; Table 1). Following this, slides were transferred to the HB (90%+ RH upon transfer). After three hours in the HB, slides 1 and 2 were removed from the HB and the mass of each was determined on the analytical balance outside of the glovebox (average of three measurements; Table S1). Since it was possible that water may have condensed on the foil (and thus added mass to the sample slide; evidence of this is in Table S1), each slide was then placed in a glassware oven at 50 °C for five minutes. It was expected that this temperature and exposure time would not be enough to remove water from the crystal structure of hydrated MgCl_2_ but would be sufficient to dry any accumulation of water on the outer surface of the foil (MgCl_2_·*n*H_2_O with *n* $\leq$ 6 decompose at temperatures at or above 69 °C with decomposition temperature increasing with decreasing *n*) (Huang et al. 2011). Following this, the average total mass of each sample (1-2) was then determined again (from three measurements taken outside of the glovebox) and the corresponding *M*_aws_ was subtracted to determine the mass of the salt after exposure to the HB; these masses are presented in Table S1. Immediately following this each sample was transferred to a labeled scintillation vial and placed in a vacuum desiccator following the procedure outlined in section 2.1.5. The same procedure was completed for sample slides 3 and 4 after six hours of exposure in the HB. Once all four samples had successfully been transferred into scintillation vials they were then moved back into the glovebox (procedure outlined in section 2.1.5). These samples were then used for X-ray diffraction experiments following the procedure outlined in the section 2.1.6 of the main text; these data can be found in Table S2 and Figure S6). Note, the samples used for the mass-gain experiments are four samples that are distinct from those discussed in section 2.2.5.

*Table S1 Results and Discussion:*

As can be seen in Table 1, the MgCl_2_ inside protected sample slides exposed to the HB for three hours had no appreciable mass-gain from this exposure (Slide 1 = -0.13%; Slide 2 = +013%). The same is true for the MgCl_2_ in Slide 3 after six hours of exposure in the HB (+0.07%). These results suggest that no appreciable water vapor is transferring from the HB into the sealed sample

environment on these timescales. It is worth noting that Slide 4 did exhibit a 0.67% increase in mass after the six-hour exposure which may be a result of a few things. First, aluminum foil did not fully cover the carbon tape applied to the edges of the microscopy slide which may have resulted in water being introduced into the tape and remaining there after the 5-minute exposure

at 50 °C. Second, it is possible that a pinhole could have been in the foil and thus introduced water vapor to the MgCl_2_ (this possibility, as well as solutions to prevent it, are discussed in the last section of the main manuscript). Lastly, it is not unreasonable to assume that the discrepancy is due to the precision of the instrument considering the 95% confidence intervals associated with other measurements presented in Table S1. Considering the results from both the mass-gain and XRD experiments, we believe that the preparation method outlined above effectively protected MgCl_2_ from ambient moisture exposure for time periods that were orders of magnitude longer than what would be required for sample transfer (ca. < 30 seconds) into the Leica VCT500.

| **Table S1.** Mass-Gain Data Collected Pre- and Post-HB Exposure |
| --- |
| 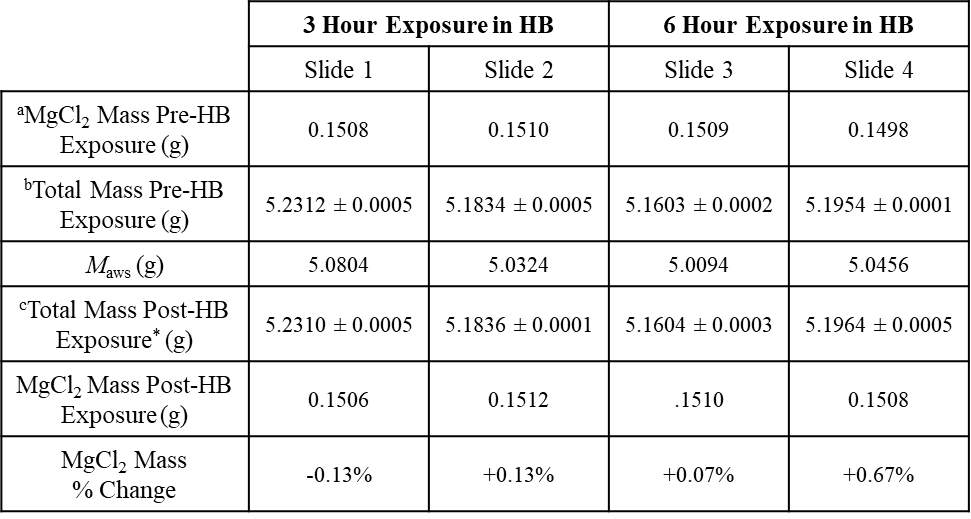. |
| Masses are (a) the result of one measurement recorded inside the glovebox, (b) the average of six measurements (three inside the glovebox and three outside of the glovebox) with a 95% confidence interval, and (c) the average of three measurements (outside of the glovebox) with a 95% confidence interval.  *Samples were transferred to glassware oven for 5 minutes at 50 °C before mass measurement. This was done to eliminate the mass of any condensed water on the outside surfaces of the foil and/or slide. |

*Table S2 Results and Discussion:*

The mass of the sample slides was attained immediately after extraction from the HB (bolded row). Following this, the sample slides were transferred to a glassware oven for 5 minutes at 50 °C to eliminate the mass of any condensed water on the outside surfaces of the foil and/or slide. This evidence suggests that mass was removed from each slide after 5 minutes at 50 °C, and we suspect that this is purely a result of condensed water on the outside surfaces of the foil and/or slide. MgCl_2_·*n*H_2_O with *n* $\leq$ 6 decompose at temperatures at or above 69 °C (decomposition temperature increases with decreasing *n*) (Huang et al. 2011), and the XRD patterns associated with these samples (Figure S3) are identical to the MgCl_2_ standard sample. If these samples had transformed into MgCl_2_·4H_2_O, which occurred after just 5 minutes of exposure for an unprotected sample (Figure 5 in the main text), then the XRD pattern should represent that transformation. It does not, which is sufficient evidence that the mass-loss after exposure to the glassware oven was not from the MgCl_2_ but instead from the outer surfaces of the foil/slide apparatus.

| 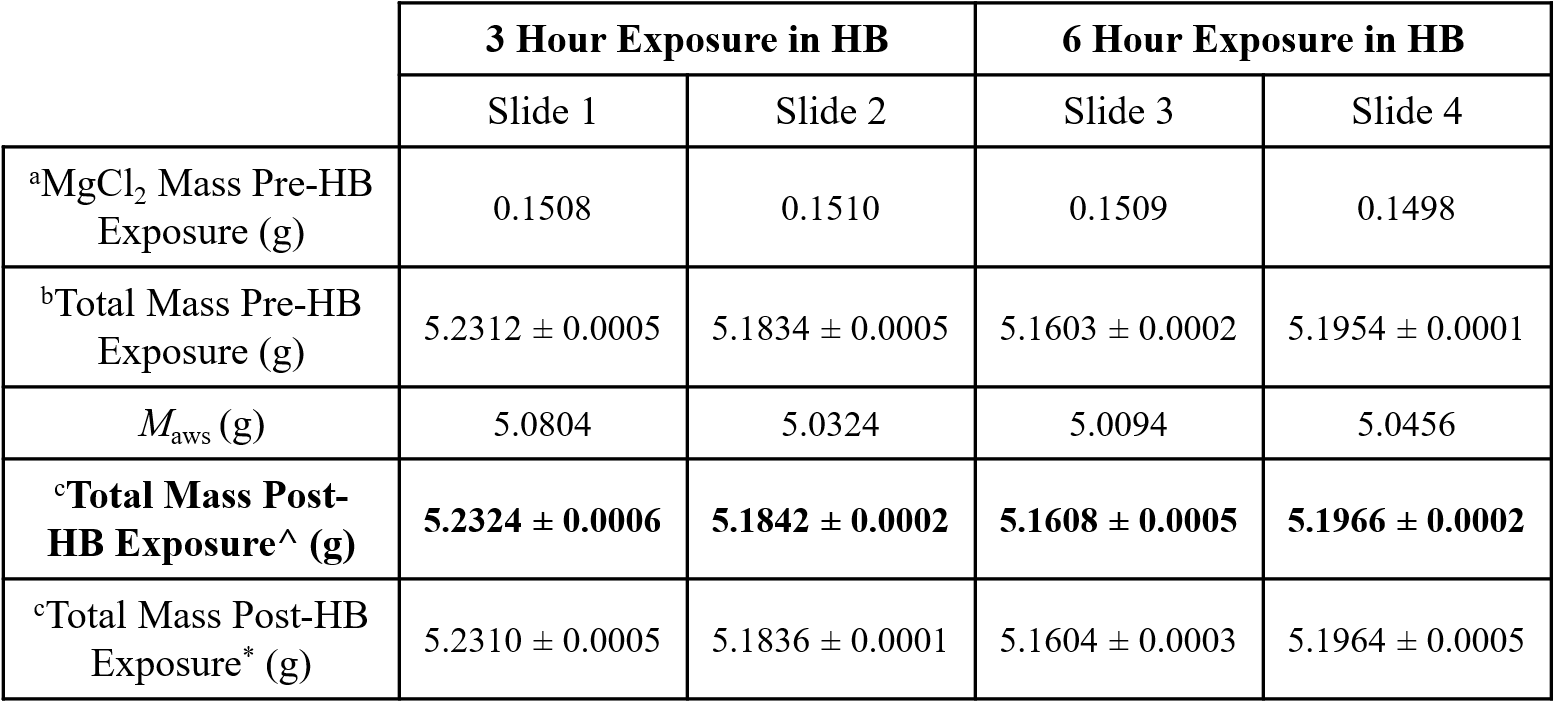**Table S2.** Mass-Gain Data Collected Pre- and Post HB Exposure |
| --- |
| . |
| Masses are (a) the result of one measurement recorded inside the glovebox, (b) the average of six measurements (three inside the glovebox and three outside of the glovebox) with a 95% confidence interval, and (c) the average of three measurements (outside of the glovebox) with a 95% confidence interval.  ^Sample mass attained immediately after removal from the HB.  *Samples were transferred to glassware oven for 5 minutes at 50 °C before mass measurement. This was done to eliminate the mass of any condensed water on the outside surfaces of the foil and/or slide. |


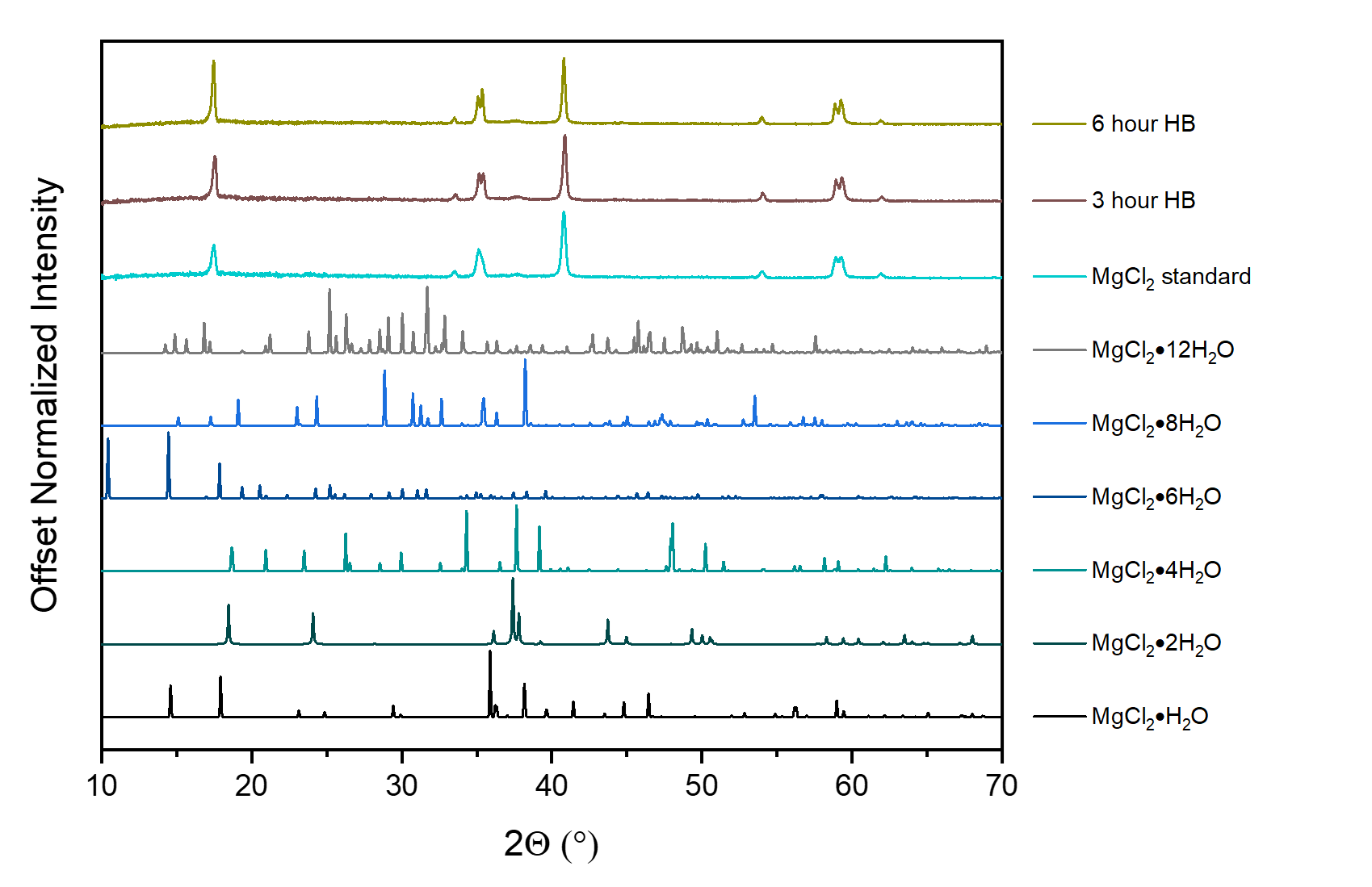


| . |
| --- |
| **Figure S6.** Powder X-ray diffraction patterns of MgCl_2_ samples exposed to the conditions of the HB for 3 or 6 hours (Slide 1 and 3 respectively) during mass gain experiments, the unexposed MgCl_2_ standard sample, and reference patterns for MgCl_2_·H_2_O (Sugimoto et al., 2007), MgCl_2_·2H_2_O (Sugimoto et al., 2007), MgCl_2_·4H_2_O (Schmidt et al., 2012), MgCl_2_·6H_2_O (Villars P & Cenzual K, 2016), MgCl_2_·8H_2_O (Hennings et al., 2013), and MgCl_2_·12H_2_O (Hennings et al., 2013). All patterns were normalized to the most intense peak and then offset on the y-axis for comparison. |

**Supporting Information Section 3: BaO Energy-dispersive X-ray Spectroscopy Data and Pt-coated BaO Micrographs**

*Energy Dispersive X-Ray Spectroscopy Data for non-coated BaO sample before and after humidity exposure.*

| _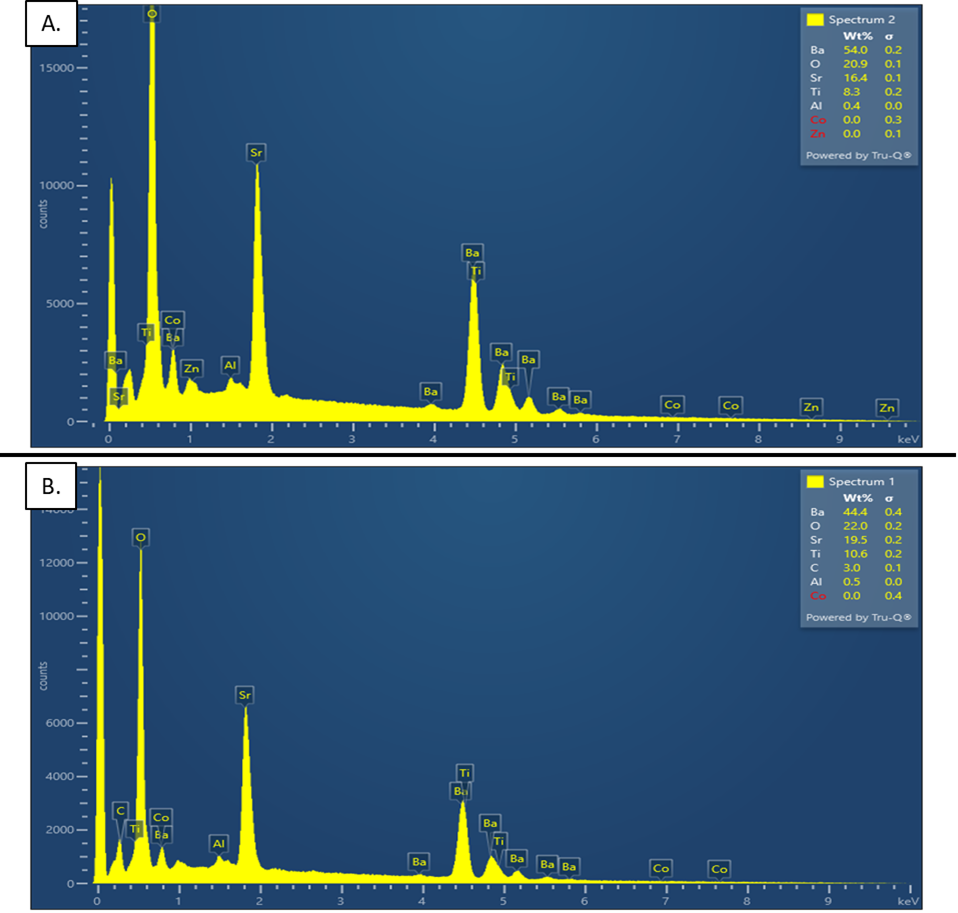_ |
| --- |
| **Figure S7.** EDS Spectrum of the protected (A) and humidity-exposed (B; 5 minutes at 100% humidity) BaO sample. Note that these EDS spectra are of the BaO sample deposited on a SrTiO_3_ substrate. |

**Figure S7 Discussion:** (**A**) As this is a BaO sample deposited on a SrTiO_3_ substrate, assume that only 25% of the O wt% is attributed to O found in the BaO material. As such, the experimental oxygen wt% attributed to BaO is 5.225% while all of the measured Ba is from the BaO material. Calculating the weight ratio (wt%) of Ba:O results in 54:5.225 or 10.24. For theoretically pure BaO, the weight ratio (wt%) of Ba:O 89.57:10.44 or 8.58. This suggests that the BaO material present is nearly pure with only a small amount of material converted to Ba(OH)_2_. **(B**) If we assume complete hydration of BaO to Ba(OH)_2_, the theoretical weight ratio (wt%) of Ba:O is 80.12:18.68 or 4.29. If we assume that the SrTiO_3_ remains pure, then 40% of the O wt% (from the EDS results) can be attributed to Ba(OH)_2_. Thus, the experimental weight ratio (wt%) of Ba:O in the material is 44.4:8.8 or 5.05—this result suggests complete conversion of BaO to Ba(OH)_2_ within 5 minutes of exposure to a 100% humid environment.

*SEM micrographs of protected BaO material sputter-coated with 2 nm Pt.*

| 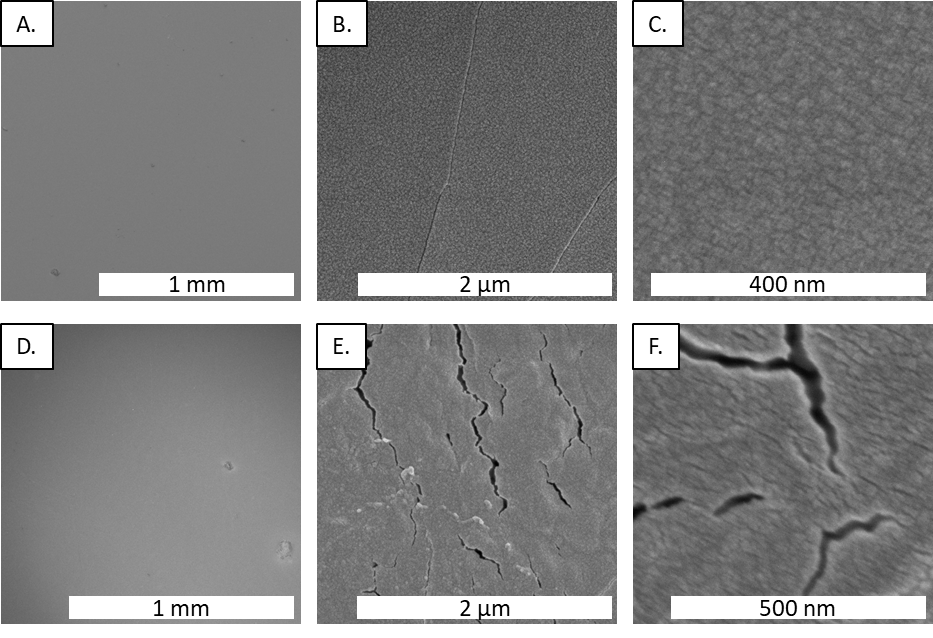 |
| --- |
| **Figure S8.** (A-C) SEM micrographs of protected BaO material sputter-coated with 2 nm Pt. Note that the surface of the material appears smooth even at high magnification. (C) The sputter coating layer can be seen deposited on top of the BaO film. |

**References**

Hennings, E., H. Schmidt, and W. Voigt. 2013. “Crystal structures of hydrates of simple inorganic salts. I. Water-rich magnesium halide hydrates MgCl2·8H2O, MgCl 2·12H2O, MgBr2·6H2O, MgBr2·9H2O, MgI2·8H2O and MgI2·9H2O.” *Acta Crystallographica Section C: Structural Chemistry* 69, no. 11: 1292–1300.

Huang, Q., G. Lu, J. Wang, and J. Yu. 2011. “Thermal decomposition mechanisms of MgCl2·6H2O and MgCl2·H2O*. Journal of analytical and Applied Pyrolysis* 91, no. 1 :159–164. <https://doi.org/10.1016/j.jaap.2011.02.005>

Schmidt, H., E. Hennings, and W. Voigt. 2012. “Magnesium Chloride Tetrahydrate, MgCl_2_·4H_2_O.” *Acta Crystallographica. Section C* 68, no. 1: i4–i6.

Sugimoto, K., R. E. Dinnebier, and J. C. Hanson. 2007. “Structures of Three Dehydration Products of Bischofite From In Situ Synchrotron Powder Diffraction Data (MgCl_2_·nH_2_O; *n*= 1, 2, 4).” *Acta Crystallographica. Section B* 63, no. 2: 235–242.

Villars, P., and K. Cenzual. 2016. “MgCl_2_·6H_2_O (MgCl_2_[H_2_O]6) Crystal Structure.” In *Inorganic Solid Phases* (P Villars and K. Cenzual, Eds.). Springer Materials.
